# Proximal tubule-derived exosomes contribute to mesangial cell injury in diabetic nephropathy via miR-92a-1-5p transfer

**DOI:** 10.1186/s12964-022-00997-y

**Published:** 2023-01-13

**Authors:** Yi-Chun Tsai, Mei-Chuan Kuo, Wei-Wen Hung, Ping-Hsun Wu, Wei-An Chang, Ling-Yu Wu, Su-Chu Lee, Ya-Ling Hsu

**Affiliations:** 1 School of Medicine, College of Medicine, Kaohsiung Medical University, Kaohsiung, Taiwan; 2 Division of General Medicine, Kaohsiung Medical University Hospital, Kaohsiung Medical University, Kaohsiung, Taiwan; 3 Division of Nephrology, Kaohsiung Medical University Hospital, Kaohsiung Medical University, Kaohsiung, Taiwan; 4 Center for Liquid Biopsy and Cohort Research, Kaohsiung Medical University, Kaohsiung, Taiwan; 5 Drug Development and Value Creation Research Center, Kaohsiung Medical University, Kaohsiung, 807 Taiwan; 6 Division of Endocrinology and Metabolism, Kaohsiung Medical University Hospital, Kaohsiung Medical University, Kaohsiung, Taiwan; 7 Division of Pulmonary and Critical Care Medicine, Kaohsiung Medical University Hospital, Kaohsiung Medical University, Kaohsiung, Taiwan; 8 Graduate Institute of Clinical Medicine, College of Medicine, Kaohsiung Medical University, Kaohsiung, Taiwan; 9 Graduate Institute of Medicine, College of Medicine, Kaohsiung Medical University, 100 TzYou 1st Road, Kaohsiung, 807 Taiwan

**Keywords:** Exosome, miR-92a-1-5p, Mesangial cell, Proximal tubular epithelial cell, Myofibroblast transdifferentiation, ER stress, Diabetic nephropathy

## Abstract

**Background:**

Diabetic nephropathy (DN) is an increasing threat to human health and regarded to be the leading cause of end-stage renal disease worldwide. Exosomes delivery may play a key role in cross-talk among kidney cells and the progression of DN. However, the mechanisms underlying exosomes in DN remain unclear.

**Methods:**

The cross-disciplinary study, including in vivo, in vitro, and human studies was conducted to explore the cross-talk between proximal tubular epithelial cells (PTECs) and mesangial cells (MCs) in DN. We purified exosome from PTECs treated with high glucose and db/db mice and assessed their influences in the pathologic change of MCs and downstream signal pathway. Healthy individuals and type 2 diabetic patients were enrolled to examine the role of exosomes in clinical applications.

**Results:**

High glucose stimulated PTECs to secrete exosomal miR-92a-1-5p, which was taken-up by glomerular MCs, inducing myofibroblast transdifferentiation (MFT) in vitro and in vivo. PTEC-released exosomal 92a-1-5p decreased reticulocalbin-3 expression, leading to endoplasmic reticulum (ER) stress by downregulating genes essential for ER homeostasis including calreticulin and mesencephalic astrocyte-derived neurotrophic factor. Treatment with miR-92a-1-5p inhibitor ameliorated kidney damage in db/db mice with DN. Urinary miR-92a-1-5p could predict kidney injury in type 2 diabetic patients.

**Conclusions:**

PTEC-derived exosomal miR-92a-1-5p modulated the kidney microenvironment in vivo and in vitro models, which altered ER stress and MFT in MCs resulting in DN progression. Further blocking miR-92a-1-5p epigenetic regulatory network could be a potential therapeutic strategy to prevent the progression of DN.

**Video Abstract**

**Supplementary Information:**

The online version contains supplementary material available at 10.1186/s12964-022-00997-y.

## Plain English Summary

Diabetic nephropathy (DN) has been the leading cause of end-stage renal disease worldwide. Exosomes play a principle role in cross-talk of kidney cells and further affect the onset or progression of DN. This study firstly demonstrated the communication between proximal tubular epithelial cells (PTECs) and mesangial cells (MCs) through exosome transmission. PTEC-released exosomal 92a-1-5p induced endoplasmic reticulum stress and epithelial-mesenchymal transition in MCs through reticulocalbin-3 modulation. Kidney damage was rescued in DN mice after treatment with miR-92a-1-5p inhibitor. Moreover, urinary exosomal miR-92a-1-5p could predict DN progression in type 2 diabetic patients. These findings prove the impact of exosomal miR-92a-1-5p on pathophysiologic mechanisms and its potential use in clinical care and prediction of DN.

**Supplementary Information:**

The online version contains supplementary material available at 10.1186/s12964-022-00997-y.

## Background

Taiwan has the highest prevalence and incidence of end stage renal disease (ESRD) in the world [1]. One in ten Taiwanese has chronic kidney disease (CKD), which is one of the ten major causes of mortality in Taiwan [1]. Consequently, CKD and its complications place a huge burden on the health insurance system. Diabetes mellitus (DM) is an increasing threat to human health and regarded as an important public issue [2]. Diabetic nephropathy (DN) is the leading cause of ESRD worldwide, accounting for 40–50% of all ESRD patients [3]. Thus, if the onset or progression of DN can be avoided, the incidence of ESRD may be ameliorated.

The pathophysiologic mechanisms of DN are complicated. Cross-talk among different types of kidney cells mediates changes in the renal microenvironment [4]. High glucose (HG) can lead to proximal tubular epithelial cells (PTECs) injury [5], and proximal tubular changes occur early in the diabetic kidney. Mesangial cells (MCs) are essential in maintaining the integrity of the glomerular structure and regulating glomerular filtration [6]. Retrograde trafficking between proximal tubules and the glomerulus has been demonstrated [7], in which nicotinamide mononucleotide released by PTECs diffuses to the glomerulus to induce podocyte foot process effacement and albuminuria [8], and proximal tubular injury also promote more extensive glomerular injury [9]. Taken together, these findings indicate that cross-talk between proximal tubules and glomerulus regulates the DN development. However, the signal communication between PTECs and MCs remains largely unknown.

Extracellular vesicles (EVs) are a heterogeneous population of microparticles released by living cells. EVs, typically composed of two primary types (exosomes and microvesicles), are regarded to be novel vectors for cell–cell communication [10]. Exosomes are small plasma membrane-derived vesicles with a diameter of 30–200 nm which carry a variety of biomolecules arising from the cell of origin [10, 11]. The importance of exosomes has been demonstrated in kidney pathophysiology [12]. Whether exosomes derived from PTECs retrograde affect glomerular cells, further resulting in worsening DN remains unknown. Understanding exosome production, the molecules they carry, and their interactions within kidney is useful to clarify the pathogenesis of kidney disease, and lead to the development of new target therapies and prognostic biomarkers for kidney disease progression. Therefore, the aim of this cross-disciplinarily study was to investigate the role of cell–cell communication between PTECs and MCs through exosomal delivery on changes in the microenvironment of DN.

## Materials and methods

### Cell culture

PTECs of a type 2 diabetic (T2D) patient and a normal individual (Lonza Walkersville Inc., MD, USA) were cultured in Clonetics™ REGM™ BulletKit™. Human PTECs (ATCC PCS-400-010) were cultured in renal epithelial cell basal medium (ATCC PCS400030TM) plus 0.5% fetal bovine serum (FBS). Human kidney-2 (HK-2) cells (ATCC®CRL-2190) were culture in keratinocyte serum free medium plus 2% FBS. Cells were treated with normal glucose (NG, 5.5 mM) and HG (25 mM) for the indicated times. Mouse MCs (MMCs, ATCC®CRL-1927) and human embryonic kidney (HEK) 293 cells (ATCC*®* CRL-1573™) were cultured in Dulbecco's Modified Eagle Medium (DMEM) with NG supplemented with 5% and 10% FBS respectively (Additional file 1: Fig. S1A). For the co-culture of HK-2 cells and MMCs, HK-2 cells and MMCs were cultured in upper inserts (pore size: 3 μm) and low 6 well plate respectively for a 48 h culture.

### Cell isolation from mice

Six-week-old, pathogen-free male C57BL/6 J mice, db/m mice and db/db mice (T2D model) [13] were purchased from the National Laboratory Animal Center in Taiwan. All animal experiments in this study were approved by Kaohsiung Medical University and Use Committee (No. 107107). Isolation and primary culture of mice PTECs and MCs, which were excised from 10 to 12 kidneys from mice at 12th week, were performed as previous reports [14]. Aquaporin 1 (AQP1) and α-smooth muscle actin (α-SMA) as well-known markers of PTECs and MCs respectively were used to identify primary PTECs and MCs (Additional file 1: Fig. S1A).

### Exosome isolation and identification

Cells were seeded at a 100 mm dish and cultured for 48 h under NG or HG condition in culture medium containing 1% exosome-free FBS (Life Technologies). The RNAs of exosomes derived from the supernatants of cells and the urine were purified using ExoQuick-TC and urine Exosome RNA Isolation Kit (NORGEN, Canada) following the manufacturer’s protocols. Exosome size was determined using nanoparticle tracking analysis [15].

### Nanoparticle tracking analysis (NTA)

Exosome size was determined using a ZetaView PMX120 (Particle Metrix GmbH, Germany). All samples were scanned in 11 cell positions and calculated using Brownian motion. ZetaView software 8.05.10 was used to export the final data and statistics. Scattered mode and fluorescence mode were used to detected fluorescent signals of nano particles with a laser at 488 nm and inserted long wave-pass filter cut-off at 500 nm.

### Fluorescence imaging of exosomes

Exosomes derived from HK-2 cells cultured were labelled PKH26 (Sigma-Aldrich), and then incubated with MMCs in NG condition for 3 h. After washing, MMCs were stained with Calcein-AM (Life Technologies) for 30 min, and pictures taken using a Nikon inverted fluorescence microscope (Eclipse TE200 microscope).

### Transmission electron microscopy (TEM)

Cells were fixed in buffer (2% paraformaldehyde and 2.5% glutaraldehyde) for 2 h for observation of endoplasmic reticulum (ER) structure. The samples were treated with 2% osmium tetroxide buffer, and placed in 0.5% aqueous uranyl acetate Sections were washed with 50% alcohol, then dehydrated in a graded series of ethanol concentrations. After being embedded in Eponate, serial sections were cut and transferred to formvar-coated slot grids and imaged on a TEM (H-7600, Hitachi, Tokyo, Japan).

### RNA sequencing and quantitative real time PCR (Q‐PCR) analyses

The RNA-sequcncing from harvested cells was performed by Welgene Biotechnology Company (Welgene, Taipei, Taiwan) [15]. The differentially expressed miRNAs of primary PTECs between the T2D patient and normal individual were defined at > twofold change and > 10 reads per million. The differentially expressed mRNAs between HK-2 cells transfected with either RCN3 siRNA (20 nM) or NC siRNA (20 nM) under NG were defined at > twofold change and > 0.3 fragments per kilobase of transcript per million. Then, we performed bioinformatics analysis and Q-PCR as previously described [15]. Total RNA from cells and exosomes was isolated using TRIzol and TRIzolLS Reagent (ThermoFisher Scientific, CA, USA), respectively. cDNA was conversed from total RNA using an oligo (dT) primer and reverse transcriptase (Takara, Shiga, Japan) following standard protocols. miRNAs were prepared using the Mir-X™ miRNA First Strand Synthesis Kit (Clontec). mRNA levels were determined using real-time analysis with SYBR Green on a QuantStudio 3 real-tme PCR system (ThermoFisher Scientific). The relative expression of a specific mRNA and miRNA in cell lysate were normalized to GAPDH or U6, respectively. miRNA in cell-secreted or urinary exosomes was normalized with spike-in control cel-miR-39 (Exiqon, Vedbaek, Denmark) and then compared with a reference sample. All primers used are listed in Additional file 2: Table S1.

### Transient transfection

miR-92a-1-5p mimic (100 nM), miR-negative control of mimic (miR-NC, 100 nM), miR-92a-1-5p inhibitor (50 nM), and miR-negative control of inhibitor (anti-miR-NC, 50 nM) (GE Healthcare, USA), reticulocalbin-3 (RCN3) siRNA (20 nM), RCN3 cDNA plasmid (4 µg), NC cDNA plasmid (4 µg), calreticulin (CALR) siRNA (20 nM), mesencephalic astrocyte derived neurotrophic factor (MANF) siRNA (20 nM) and NC siRNA (20 nM) were transfected into cells using LipofectamineTM RNAiMAX transfection reagent (ThermoFisher Scientific) following the manufacturer’s protocols.

### Western blot analysis

The total protein of MMCs treated with HK-2 cell-derived exosomes with 1.5 to 3 μg was extracted using RIPA (radio-immunoprecipitation assay) lysis buffer (EMD Millipore, USA). The denature protein was separated by 9–11% SDS-PAGE electrophoresis, and then transferred onto a PVDF membrane following blocking and immunoblotting with specific primary and secondary antibodies. Antibodies against heat shock proteins 70 (HSP70), tumor susceptibility gene 101 (Tsg101), CD9, CD63, CD81, AQP1, α-SMA, N-cadherin, vimentin, E-cadherin, collagen I, RCN3, activating transcription factor 6 (ATF6), phospho-protein kinase r-like endoplasmic reticulum kinase (p-PERK), PERK, phospho-inositol-requiring transmembrane kinase/endoribonuclease 1α (p-IRE1α), IRE1α, C/EBP homologous protein (CHOP), CALR, MANF, and GAPDH were used (Additional file 3: Table S2). The signals of blots were captured using Proteinsimple + Fluorchem Q system (Alpha Innotech, USA). Densitometry of the blots was calculated using Image J software (USA).

### 3′UTR luciferase reporter assay

As numerous studies [16, 17], human embryonic kidney (HEK) 293 cells (1 × 104/well) had been used to be co-transfected with pGL3-RCN3-2–3′ untranslated region (3′UTR) luciferase plasmid/pRL-TK Renilla (8:1) or pGL3-RCN3-3′UTR mutated (MT) luciferase plasmid/pRL-TK Renilla (8:1) with miRNA mimics (control mimic or miR-92a-1-5p mimic) using DharmaFECT Duo Transfection Reagent (ThermoFisher Scientific) for 48 h. The activities of firefly and Renilla luciferase were then quantified using the Dual-Glo® Luciferase Assay System (Promega, USA).

### Animal experiments

miR-92a-1-5p antagomir (n = 4) or NC antagomir (n = 4) were administered to db/db mice at 8th week intravenously via tail vein at doses of 5ug/mice three times per week for 3 weeks. The efficacy of oligonucleotides administration was confirmed in kidney afterfluorescence-labeled oligonucleotidesinjection (Additional file 1: Fig. S1B), and miR-92a-1-5p antagomir did not affect liver function (Additional file 1: Fig. S1C). Body weight and blood glucose levels were monitored, and 24-h urine of mouse were collected every week. After the final injection, the mice were sacrificed and serum was collected, and then, the kidneys were harvested and fixed in 4% paraformaldehyde.

### Immunohistochemistry stain (IHC) stain

The mesangial matrix index (%) was expressed as mesangial matrix area/tuft area × 100% using Periodic Acid-Schiff (PAS) stain according to the manufacturer’s protocols (Sigma). Antibodies used for IHC were listed in Additional file 3: Table S2. Stained kidneys were observed using Leica microscope (ICC50 HD, USA) and quantification was performed using the IHC Profiler Plugin of ImageJ Software.

### Human study participants

Forty-four T2D patients with estimated glomerular filtration rate (eGFR) ≥ 30 ml/min/1.73m^2^ and 36 healthy volunteers with similar age to our T2D patients from communities were invited. Demographic and medical data were obtained from medical records and interviews with patients. Blood and urine samples were taken after a 12 h fast for biochemistry studies at enrollment, and stored in a − 80 °C freezer. Kidney sections were obtained from DN patients scheduled for biopsies and upper tract urothelial carcinoma (UTUC) patients receiving nephrectomy. This study was approved by the Institutional Review Board of Kaohsiung Medical University Hospital (KMUHIRB-G(I)-20150044). All participants provided written informed consent in accordance with the Declaration of Helsinki.

### Measurement of kidney functions and injury in mice and human

Levels of urinary albumin were assessed using the immunoturbidimetric assay with Tina-quant Albumin Gen.2 (ALBT2, Roche, USA). Levels of kidney injury molecule 1 (KIM-1) and neutrophil gelatinase-associated lipocalin (NGAL) in the urine of mice and human were measured using enzyme-linked immunosorbent assay (ELISA) kit and Magnetic Luminex® Assay respectively. All urinary parameters were corrected by urinary creatinine (Cr). Concentrations of serum urea nitrogen (UN) and urinary Cr were examined using the enzymatic method (Roche Diagnostics, Mannheim, Germany). Serum Cr was measured using the compensated Jaffé method in a Roche/Integra 400 Analyzer (Roche Diagnostics) [18]. eGFR was calculated using the equation of the 4-variable Modification of Diet in Renal Disease Study [19].

### Statistical analysis

Continuous variables were expressed as mean ± standard error of the mean (S.E.M) or median (25th, 75th percentile) as appropriate, and correlations among continuous variables were examined using Spearman correlation analysis. Categorical variables were expressed as percentages, and differences were tested using chi-square test. The differences in continuous variables between groups were analyzed using Student’s t-test or one-way ANOVA, followed by the post hoc test with a Tukey’s correction. Statistical analyses were conducted using SPSS version 22.0 (SPSS Inc., Chicago, Illinois) and Graph Pad Prism 9.2.0 (GraphPad Software Inc., San Diego CA, USA). Statistical significance was set at a two-sided *p*-value of < 0.05.

## Results

### Identifying the presence of PTEC-derived exosomes

**Fig. 1:**
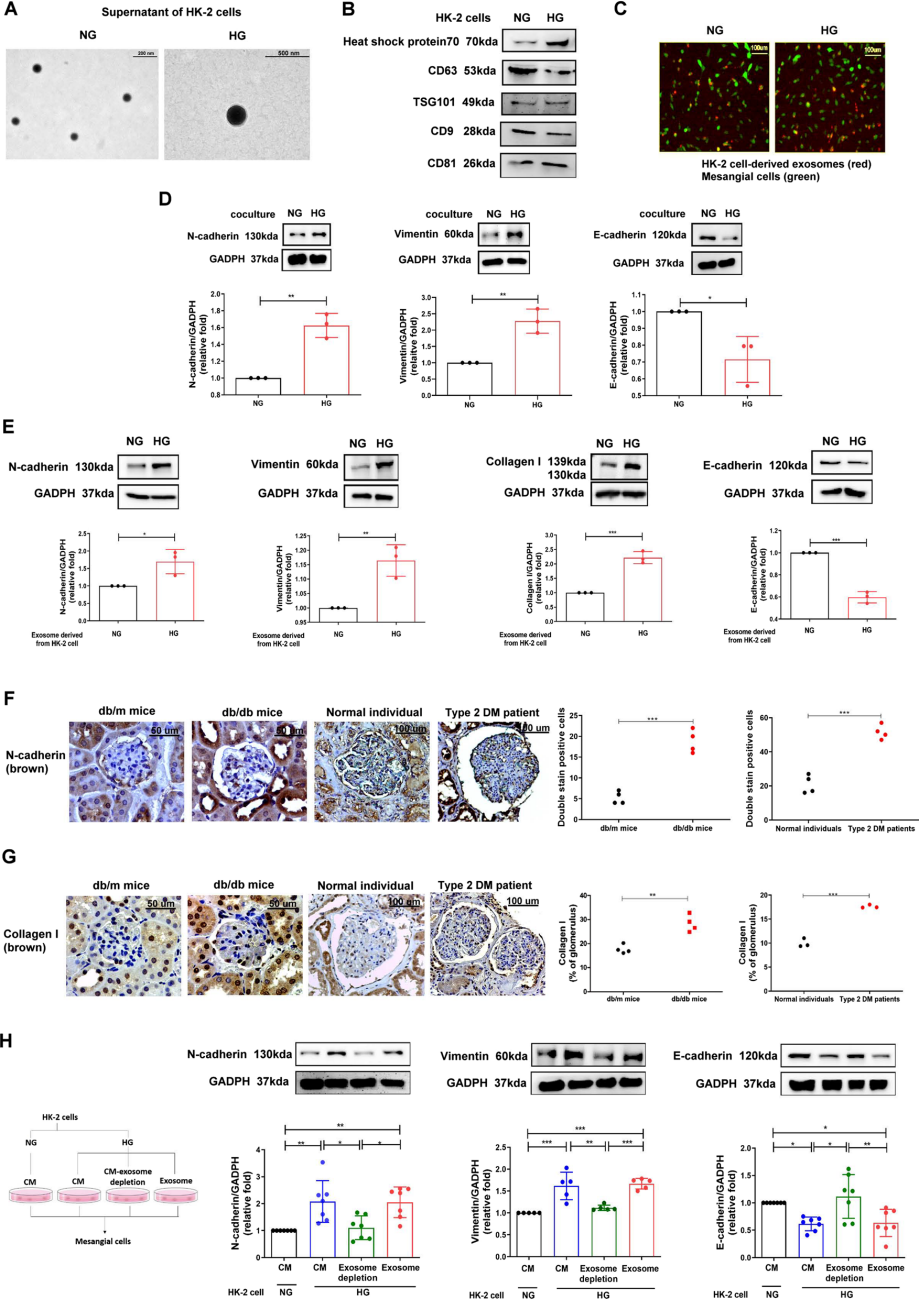
Identifying the presence of proximal tubular epithelial cell (PTEC)-derived exosomes which promoted myofibroblast transdifferentiation (MFT) in mesangial cells (MCs). **A** Transmission electron microscope (TEM) analysis revealed exosomes in the secretion of PTECs. **B** The surface markers of exosomes derived from HK-2 cells treated with normal glucose (NG, 5.5 mM) or high glucose (HG, 25 mM) using western blotting. **C** Detection of NG- or HG-treated HK-2 cell-derived exosomes uptake by mouse mesangial cells (MMCs) using immunofluorescence stain. **D** MFT markers were assessed in MMCs treated with condition medium of HK-2 cells under NG and HG conditions for 48 h using a co-culture system. **E** MFT markers were assessed in MMCs treated with exosomes derived from HK-2 cells under NG and HG conditions for 48 h (HK-2 cells: MMCs = 5:1). **F, G** The expression of N-cadherin and collagen I in kidneys of mice and humans. Kidney sections of non-diabetic db/m mice (n = 4) and diabetic db/db mice (n = 4) at 12th week, and human donors (upper tract urothelial carcinoma (UTUC)) with normal kidney function and normal glomeruli) (n = 4) and patients with DN (n = 4) were stained with N-cadherin and collagen I (brown) and α-smooth muscle actin (SMA, green). **H** MMCs were treated with condition medium (CM) derived from HK-2 cells under NG and HG conditions, HG-induced HK-2 cell CM after removing exosomes, and HG-induced HK-2 cell-derived exosomes for 48 h. Western blotting was used to measure N-cadherin, vimentin, collagen I and E-cadherin protein expression. The images quantification was performed using the IHC Profiler Plugin of ImageJ Software. The bar graph represents the mean ± S.E.M. of at least three independent experiments. *p < 0.05, **p < 0.01, ***p < 0.001 by Student’s t-test or ANOVA followed by the post hoc test with Tukey’s correction

To confirm the characteristics of exosomes, we used TEM to examine HK-2 cells and their exosomes after stimulation with NG and HG for 24 h (Fig. 1A). Western blotting revealed the presence of exosome-associated markers of HK-2 cell-released exosomes (Fig. 1B). NTA indicated that average diameters of exosomes were 183.4 ± 151.3 and 172.7 ± 87.3 nm under NG and HG respectively (Additional file 4: Fig. S2A). IHC stain revealed the presence of exosomes using CD63 staining in proximal tubules and MCs of both mice and humans regardless of the presence of DM (Additional file 4: Fig. S2B), and western blotting revealed the presence of exosome-associated markers in the exosomes isolated from HK-2 cells (Additional file 4: Fig. S2C). Immunofluorescence revealed that MMCs took up NG- or HG-treated-HK-2 cell-derived exosomes (Fig. 1C). These results suggested the presence of exosomal interactions between PTECs and MCs.

### PTEC-derived exosomes promoted myofibroblast transdifferentiation (MFT) in MCs

As MFT in MCs is one of major pathophysiologic mechanisms of glomerular injury [20], we utilized a co-culture system to mimic the communication between PTECs and MCs [21], and found MFT features, in MMCs under HG condition (Fig. 1D). HK-2 cell-derived exosomes induced elevated expression of N-cadherin and vimentin, and decreased expression E-cadherin in MMCs (Additional file 4: Fig. S2D, Fig. 1E). IHC stain revealed increased collagen I deposition (Fig. 1F) and N-cadherin levels (Fig. 1G) in MCs of glomerulus of db/db mice and the T2D patient [22]. We further explored the impact of HG-treated-HK-2 cell-derived exosomes on MMCs. Our result shows that MFT was induced in MMCs treated with HG-treated-HK-2 cell-derived CM or exosomes, but not in those treated with exosome-depleted CM obtained from HG-treated-HK-2 cells (Fig. 1H), suggesting HG stimulated PTECs to secrete exosomes, which lead to MFT in MCs.

### PTEC-derived exosomal miR-92a-1-5p induced MFT in MCs

**Fig. 2:**
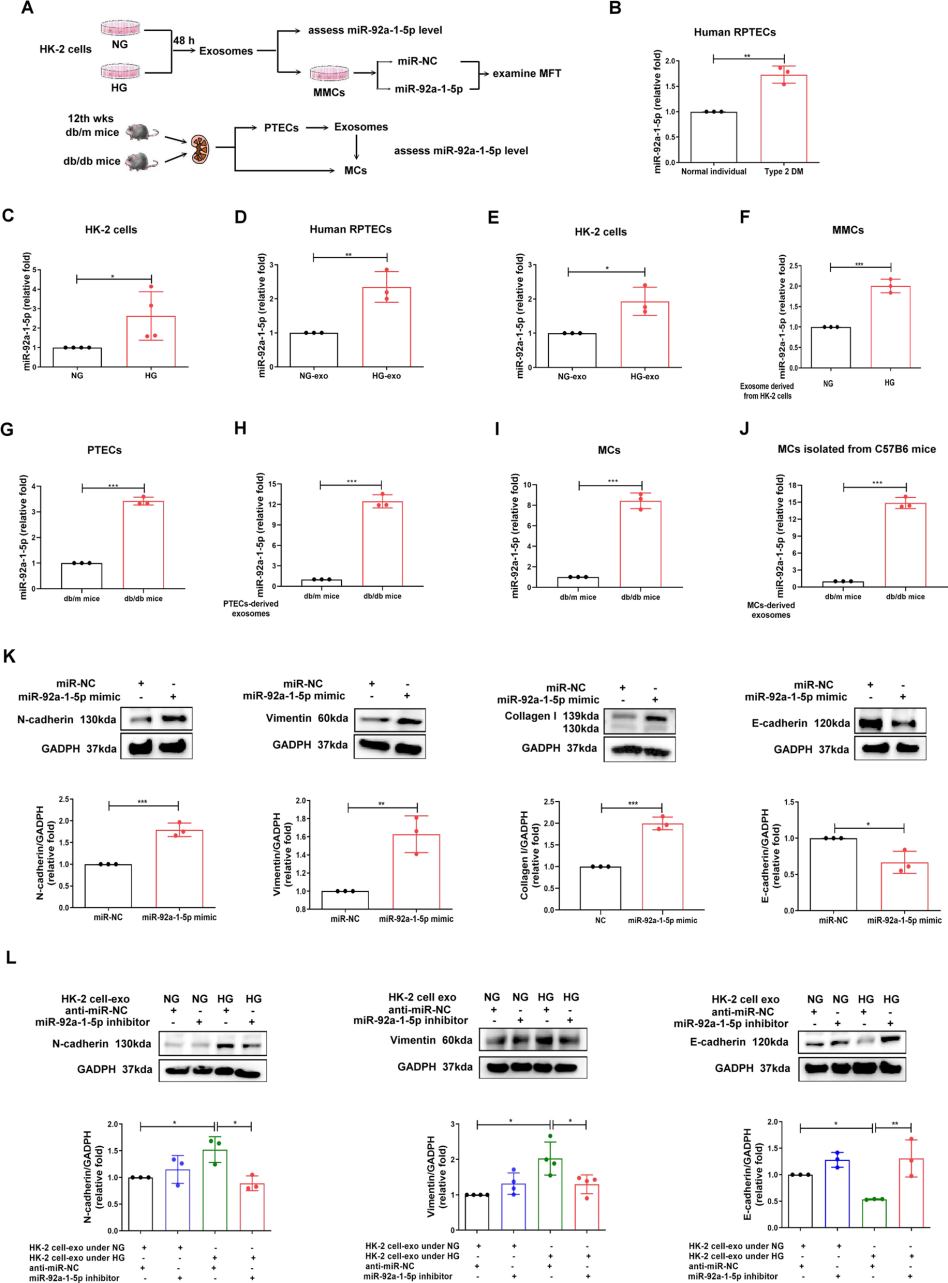
HG-induced PTEC-derived exosomes contribute to MFT in MCs through miR-92a-1-5p. **A** The flow chart of miR-92a-1-5p functional assay. **B-F** miR-92a-1-5p expression in renal PTECs (RPTECs) of a normal individual and a type 2 diabetic patient, HK-2 cells treated with NG and HG conditions for 48 h, exosomes derived from human RPTECs and HK-2 cells treated with HG for 48 h, and MMCs treated with HG-induced HK-2 cell-derived exosomes for 24 h. **G-J** miR-92a-1-5p levels in PTECs, PTEC-derived exosomes and MCs isolated from db/m mice and db/db mice and MCs isolated from C57B6 mice treated with HK-2 cell–derived from exosomes under NG and HG assessed by quantitative real-time PCR. **K** MMCs were transfected with either miR-92a-1-5p mimic (100 nM) or mimic control (miR-NC, 100 nM). After 24 h post-transfection, cells were cultured under NG condition for 24 h. **L** MMCs were transfected with miR-92a-1-5p inhibitor (50 nM) or control inhibitor (anti-miR-NC, 50 nM) for 24 h and then incubated with exosomes derived from HK-2 cells under NG or HG condition for another 48 h. N-cadherin, vimentin, collagen I and E-cadherin expressions were assessed using western blotting. The bar graph represents the mean ± S.E.M. of at least three independent experiments. RPM, read per million. *p < 0.05, **p < 0.01, ***p < 0.001 by Student’s t-test or ANOVA followed by the post hoc test adjusted with Tukey’s correction

To elucidate the potential miRNAs involved in PTEC-MC interactions after HG stimulation, the miRNA profiles of PTECs of a normal individual and a patient with T2D were established using RNA-sequencing, and analyzed using in silico websites (Additional file 5: Fig. S3A). Sixty-seven miRNAs (3 up-regulation and 64 down-regulation) with a significant two-fold change were found in PTECs of the T2D patient (Additional file 5: Fig. S3B) [23]. Of three up-regulated miRNAs, miR-378i regulated PTEC senescence of DN [23], and miR-4454 was poorly conserved, whereas miR-92a-1-5p might regulate the genes inducing kidney injury by IPA analysis (Additional file 5: Fig. S3C, Fig. 2A). The levels of miR-92a-1-5p was increased in the T2D patients’ PTECs (Fig. 2B), HG-treated-HK-2 cells (Fig. 2C), and HG-treated-HK-2 cell-derived exosomes (Fig. 2D, E). The elevated expression of miR-92a-1-5p was also found in MMCs received HG-treated-HK-2 cell-derived exosomes (Fig. 2F). db/db mice study further revealed increased expression of miR-92a-1-5p in both primary PTECs and their exosomes (Fig. 2G, H), as well as primary MCs (Fig. 2I). The level of miR-92a-1-5p level was elevated in primary MCs isolated from C57B6 mice received HG-treated-HK-2 cell-derived exosomes (Fig. 2J). Moreover, miR-92a-1-5p mimic transfection induced MFT in MMCs (Fig. 2K), and miR-92a-1-5p inhibitor transfection reversed MFT in MMCs caused by HG-treated-HK-2 cells-derived exosomes (Fig. 2L). In brief, exosomal-miR-92a-1-5p derived from HG-treated-HK-2 cells distantly regulated MFT process in MCs in both in vitro and animal models.

### Exosomes derived from HG-treated-PTECs induced endoplasmic reticulum (ER) stress in MCs

**Fig. 3:**
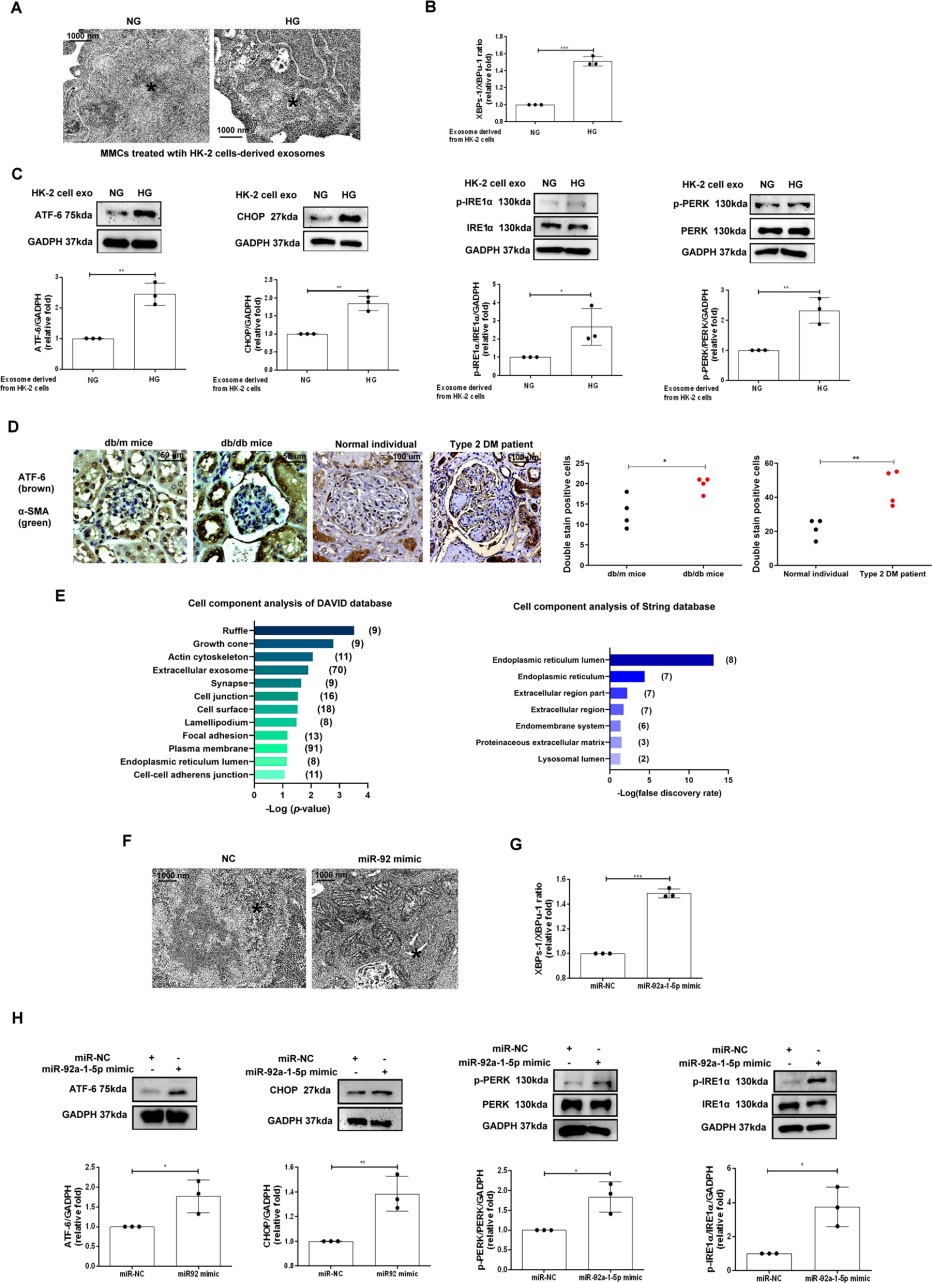
Exosomes derived from HK-2 cells treated with HG induced endoplasmic reticulum (ER) stress in MMCs through miR-92a-1-5p. **A** TEM analysis revealed ER structure of MMCs treated with NG or HG-treated HK-2 cell-derived exosomes with 10 μg for 72 h. ^*^ as ER structure. **B** qPCR was used to measure X-box binding protein 1 spliced and unspliced (XBPs-1/XBPμ-1) ratio in MMCs treated with NG or HG-treated HK-2 cell-derived exosomes for 48 h. **C** After treatment with NG or HG-treated HK-2 cell-derived exosomes for 48 h, activating transcription factor 6 (ATF-6), C/EBP homologous protein (CHOP), phospho-inositol-requiring transmembrane kinase/endoribonuclease 1α (IRE1α) and phospho-protein kinase RNA-like endoplasmic reticulum kinase (PERK) expression in MMCs were assessed by Western blotting. **D** The levels of ATF-6 in MCs of kidneys in mice and humans. The kidney sections of non-diabetic db/m mice (n = 4) and diabetic db/db mice (n = 4) at 12th week, and human donors (UTUC with normal kidney function and normal glomeruli) (n = 4) and patients with DN (n = 4) were co-stained with ATF-6 (brown) and α-SMA (green), as the marker of MCs. Arrow shows as MCs. **E** The Ontology analysis of cell component for potential genes targeted by miR-92a-1-5p using DAVID database and STRING database. **F** TEM analysis revealed ER structure of MMCs transfected with miR-92a-1-5p mimic (100 nM) or mimic control (miR-NC, 100 nM) for 24 h and then treated under NG condition for 48 h. **G** The ratio of XBPs-1/XBPμ-1 in MMCs transfected with miR-92a-1-5p mimic for 24 h and then treated under NG condition for 48 h was examined using qPCR. **H** Western blotting analysis of ATF-6, phospho-IRE1α and phospho-PERK, and CHOP expression in MMCs transfected with miR-92a-1-5p for 24 h and then treated under NG condition for 48 h. The images quantification was performed using the IHC Profiler Plugin of ImageJ Software. The bar graph represents the mean ± S.E.M. of at least three independent experiments. *p < 0.05, **p < 0.01, ***p < 0.001 by Student’s t-test

Accumulating evidence has shown that ER stress induces MFT and accelerate the progression of DN [24]. TEM showed swollen and fragmented ER structures in MMCs exposed to HG-treated-HK-2 cells-derived exosomes, suggesting that HK-2 cell-derived exosomes induced ER stress in MMCs (Fig. 3A). X-box binding protein-1 spliced and unspliced (XBPs-1/XBPμ-1) ratio was elevated in MMCs treated with HG-treated-HK-2 cell-derived exosomes (Fig. 3B). Immunoblotting revealed increased expressions of ATF6, CHOP, and p-IRE1α and p-PERK, further supporting that ER stress was induced in MMCs by HG-treated-HK-2 cells-derived exosomes (Fig. 3C). Moreover, the elevated ATF-6 expression was also observed in MCs of db/db mice and the patient with DN (Fig. 3D). Bioinformatics analysis indicated that target genes of miR-92a-1-5p locate in the ER lumen (Fig. 3E). Transfection of miR-92a-1-5p mimic in MMCs induced ER stress, which was supported by swollen and fragmented ER structures (Fig. 3F), increased XBPs-1/XBPμ-1 ratio (Fig. 3G), and other ER stress markers (Fig. 3H). Together, HG-treated-HK-2 cells-derived exosomes resulted in ER stress in MMCs through the delivery of miR-92a-1-5p.

### RCN3 as a direct target of miR-92a-1-5p contributed to MFT in MCs

**Fig. 4:**
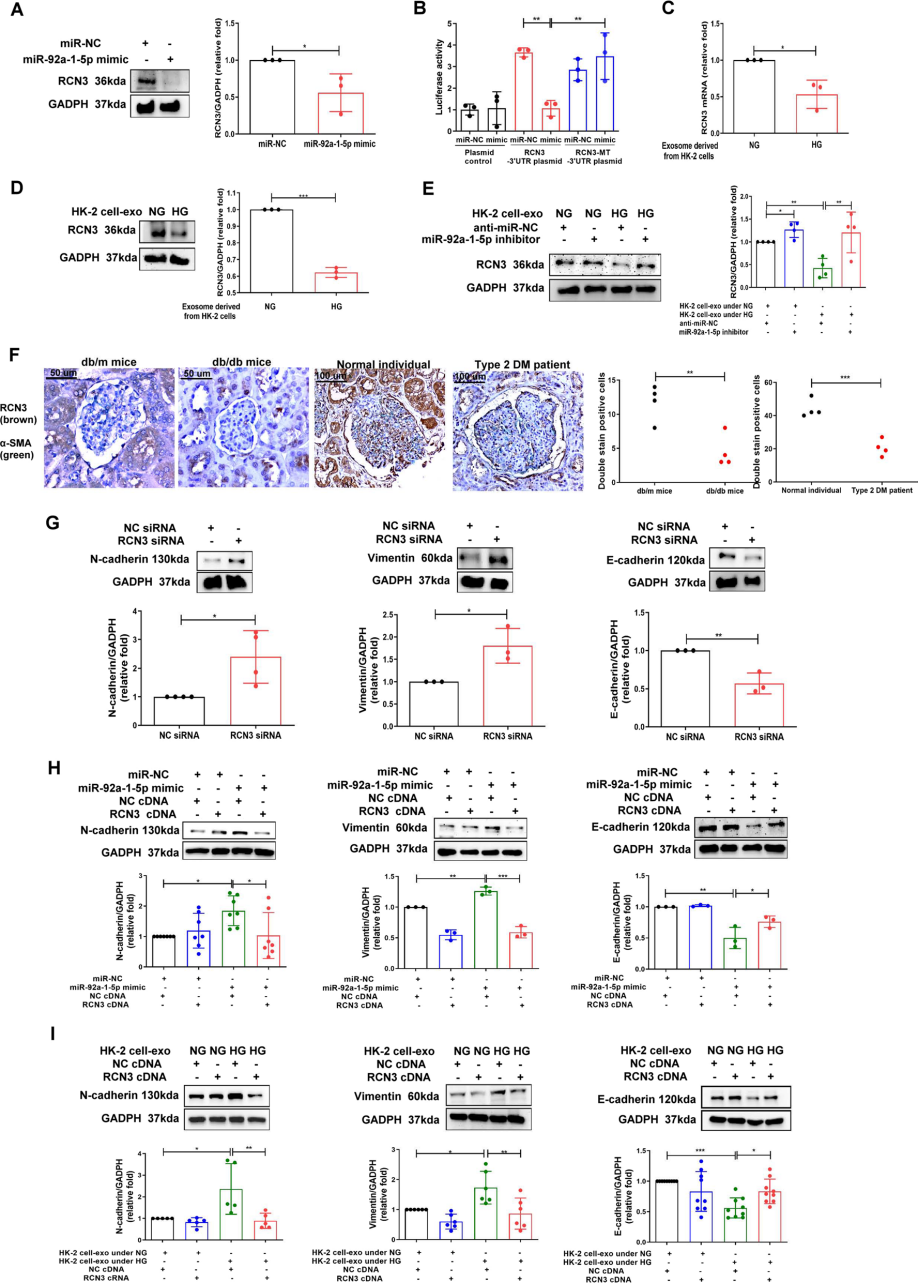
RCN3 was a direct target of miR-92a-1-5p and exosomal miR-92a-1-5p derived from HK-2 cells promoted MFT through suppressing RCN3 in MMCs. **A** RCN3 expression in MMCs after transfection with miR-92a-1-5p mimic (100 nM) or mimic control (miR-NC, 100 nM). **B** HEK 293 cells were co-transfected with pGL3-RCN3-3′UTR luciferase plasmid/pRL-TK Renilla (8:1) or pGL3- RCN3-3′UTR MT luciferase plasmid/pRL-TK Renilla (8:1) with various miRNA mimics (control mimic or miR-92a-1-5p mimic) using DharmaFECT Duo Transfection Reagent. After 48 h, both firefly and Renilla luciferase activities were quantified using the Dual-Glo® Luciferase Assay System. **C, D** RCN3 mRNA and protein expression in MMCs treated with HG-treated HK-2 cell-derived exosomes for 48 h. **E** After transfection with miR-92a-1-5p inhibitor (50 nM) or control inhibitor (anti-miR-NC, 50 nM) for 24 h, MMCs were incubated with exosomes derived from HK-2 cells under NG or HG condition. Western blotting was used to measure RCN3 protein expression. **F** The level of RCN3 in MCs of kidneys in mice and humans. The kidney sections of non-diabetic db/m mice (n = 4) and diabetic db/db mice (n = 4) at 12th week, and human donors (UTUC with normal kidney function and normal glomeruli) (n = 4) and patients with DN (n = 4) were co-stained with RCN3 (brown) and α-SMA (green), as the marker of MCs. Arrow shows as MCs. **G** MMCs were transfected with RCN3 siRNA, and 24 h after transfection, the cells were treated with NG for 48 h. **H, I** MMCs were transfected with RCN3 cDNA or control plasmid, and 24 h after transfection, the cells were treated with HG-treated HK-2 cell-derived exosomes or transfected with miR-92a-1-5p for 48 h. N-cadherin, vimentin and E-cadherin expressions were assessed using western blotting The images quantification was performed using the IHC Profiler Plugin of ImageJ Software. The bar graph represents the mean ± S.E.M. of at least three independent experiments. *p < 0.05, **p < 0.01, ***p < 0.001 by Student’s t-test or ANOVA followed by the post hoc test with Tukey’s correction

We next investigated the potential downstream targets of miR-92a-1-5p which participated in HK-2 cell-mediated ER stress and MFT in MMCs. Of all miR-92a-1-5p targets, RCN3 might contribute to ER stress and kidney injury (Additional file 6: Table S3–5). miRmap and TargetScan (version 7.1) indicated the 3′UTR of RCN3 contained the species–conserved binding sites for miR-92a-1-5p seed sequences (Additional file 7: Fig. S4A, Fig. 3B). Transfection of miR-92a-1-5p mimic decreased the expression of RCN3 protein in MMCs (Fig. 4A), and luciferase receptor assay showed that the miR-92a-1-5p directly bound to 3′UTR of RCN3 (Fig. 4B). HG-treated-HK-2 cells-derived exosomes decreased mRNA and protein level of RCN3 in MMCs (Fig. 4C, D). Conversely, transfection of miR-92a-1-5p inhibitor increased RCN3 levels in MMCs, and also reversed RCN3 downregulation induced by HG-treated-HK-2 cells-derived exosomes (Fig. 4E). The expression of RCN3 was decreased in MCs of db/db mice and the patient with DN (Fig. 4F).

The role of miR-92a-1-5p-RCN3 axis in MFT of MMCs was examined using loss- and gain-of-function models. Silencing RCN3 by siRNA transfection and overexpression of RCN3 by cDNA transfection were performed in MMCs (Additional file 8: Figs. S5A and S5B). Silencing RCN3 increased MFT (Fig. 4G). Conversely, the effects of miR-92a-1-5p transfection (Fig. 4H) and HG-treated-HK-2 cells-derived exosomes (Fig. 4I) on MFT induction in MMCs were prevented while RCN3 overexpressed in MMCs. Thus, RCN3 is the target of miR-92a-1-5p derived from HG-treated-HK-2 cells.

### RCN3 downregulation induced ER stress in MCs

**Fig. 5:**
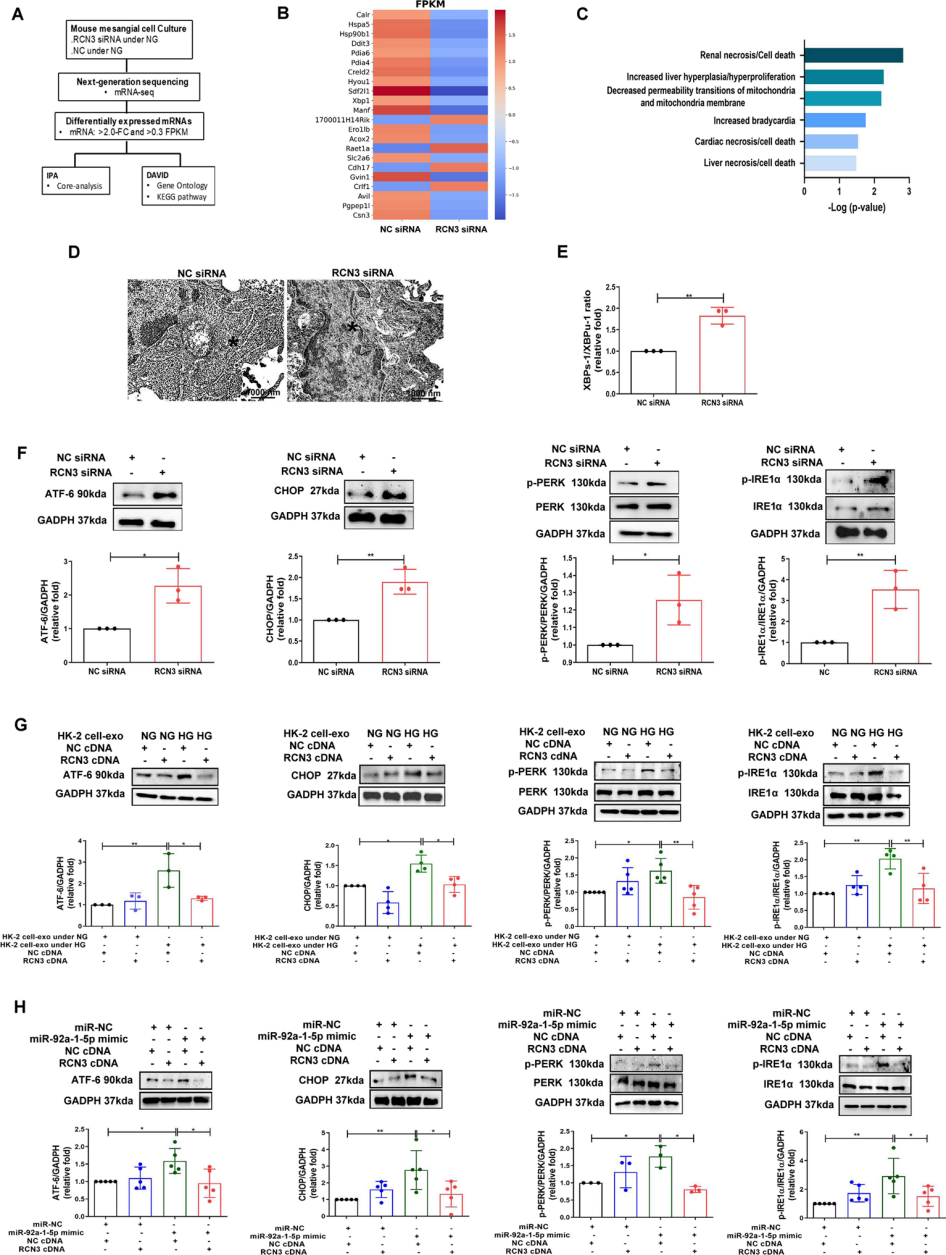
HG-induced HK-2 cell-derived exosomes promote ER stress in MMCs through miR-92a-1-5p-RCN3 pathway. **A** Flowchart of identification of potential mRNAs from MMCs transfected with RCN3 siRNA (20 nM) and normal control (NC) using NGS and following bioinformatics analysis. **B** The heat map revealed differentially expressed mRNAs from MMCs transfected with RCN3 siRNA and NC with Z-score values. **C** Signaling pathway analysis of mRNAs related to RCN3 knockdown in MMCs according to IPA core analysis. **D** Transmission electron microscope analysis revealed ER structure of MMCs transfected with RCN3 siRNA for 24 h and then treated under NG condition for 48 h. ^*^as ER structure. **E** The XBPμ-1/XBPs-1 ratio in MMCs transfected with RCN3 siRNA for 24 h and then treated under NG condition for 48 h was assessed using qPCR. **F** MMCs transfected with RCN3 siRNA for 24 h and then treated under NG condition for 48 h. **G, H** MMCs were transfected with RCN3 cDNA, and 24 h after transfection, the cells were treated with HG-treated HK-2 cell-derived exosomes or transfected with miR-92a-1-5p for 48 h. Western blotting analysis of ATF-6, CHOP, phospho-PERK and phospho-IRE1α expression. The bar graph represents the mean ± S.E.M. of at least three independent experiments. *p < 0.05, **p < 0.01, ***p < 0.001 by Student’s t-test or ANOVA followed by the post hoc test with Tukey’s correction

To further investigate the role of RCN3 in ER stress induction, transcriptome of RCN3-silencing MMCs was established by RNA-sequencing (Fig. 5A). Twenty-two mRNAs (4 up-regulation and 18 down-regulation) with a significant twofold change were found in MMCs with RCN3 knockdown (Fig. 5B). These genes were involved in kidney injury (Fig. 5C), and also participated in ER stress according to bioinformatics analysis (Additional file 7: Figs. S4C–E). TEM showed swollen and fragmented ER structures (Fig. 5D), and elevated ER stress markers (Fig. 5E, F) were found in MMCs after RCN3 knockdown. Overexpression of RCN3 prevented the induction of ER stress in MMCs exposed to HG-treated-HK-2 cell-derived exosomes or miR-92a-1-5p transfection (Fig. 5G, H). Silencing RCN3 may be associated with kidney injury through ER stress.

### CALR and MANF as downstream factors of RCN3

**Fig. 6:**
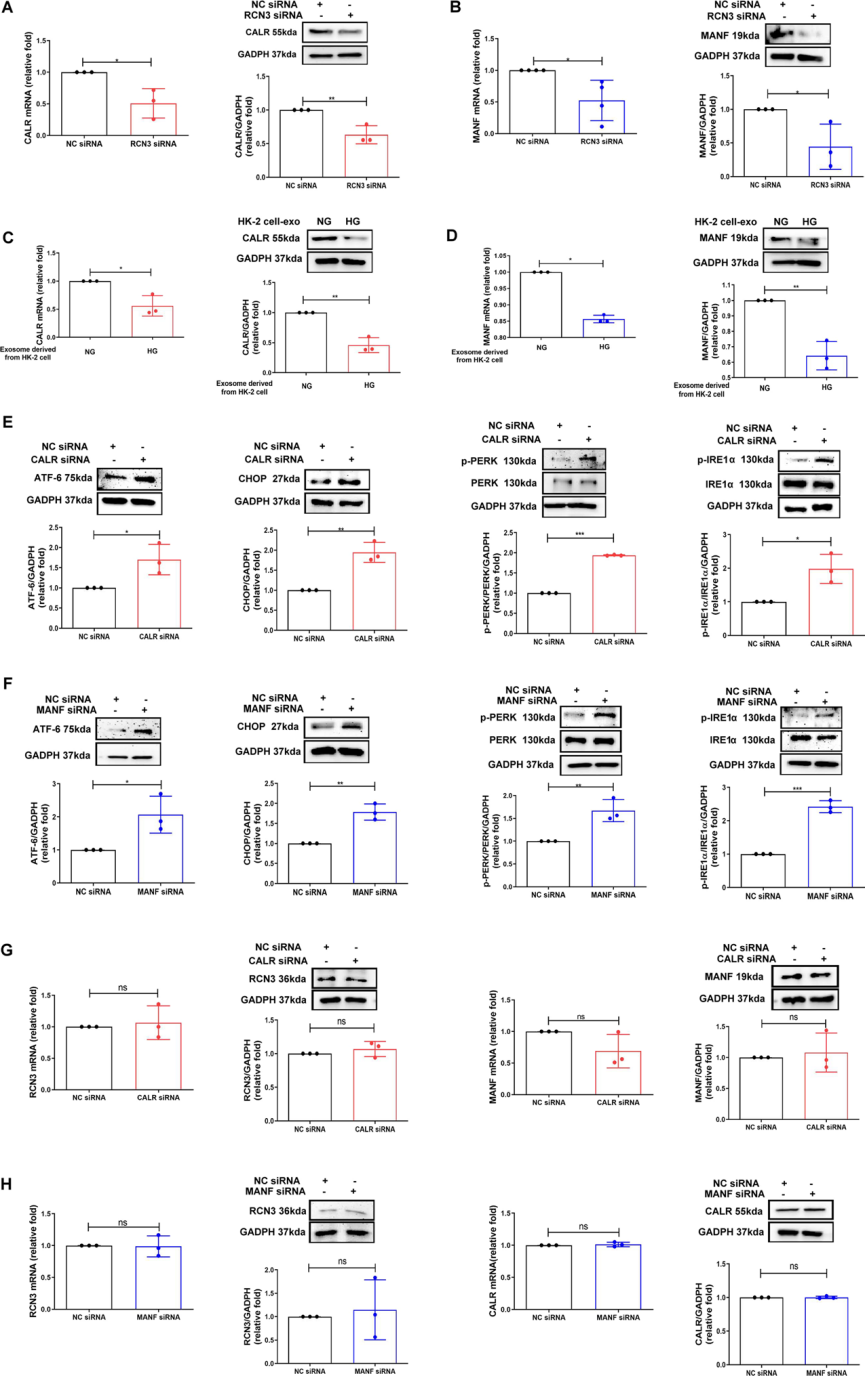
Silencing RCN3 induces ER stress in MMCs through calreticulin (CALR) and mesencephalic astrocyte derived neurotrophic factor (MANF) suppression. **A**, **B** MMCs were transfected with RCN3 siRNA (20 nM) for 24 h, and then treated under NG condition for 48 h. CALR and MANF mRNA and protein expression were measured. **C, D** MMCs were treated with HG-treated HK-2 exosomes for 48 h. CALR and MANF mRNA and protein expressions were examined. **E, F** ATF-6, CHOP, phospho-PERK and phospho-IRE1α protein expressions were assessed in MMCs transfected with CALR or MANF siRNA for 24 h, and then treated under NG condition for 48 h. **G, H** RCN3 mRNA and protein expression were assessed in MMCs transfected with CALR or MANF siRNA for 24 h, and then treated under NG condition for 48 h. MANF mRNA and protein expression were measured in MMCs transfected with CALR siRNA for 24 h, and then treated under NG condition for 48 h. CALR mRNA and protein expression were measured in MMCs transfected with MANF siRNA for 24 h, and then treated under NG condition for 48 h. mRNA and protein level were measured using qPCR and Western blotting respectively. The bar graph represents the mean ± S.E.M. of at least three independent experiments. *p < 0.05, **p < 0.01, ***p < 0.001 by Student’s t-test

We further evaluated the downstream pathway of RCN3 mediating ER stress. Two ER structure factors CALR and MANF were decreased in MMCs after RCN3 knockdown (Fig. 6A, B, Additional file 9: Table S6), as shown in RNA-sequencing. HG-treated-HK-2 cells-derived exosomes reduced mRNA and protein expressions of CALR (Fig. 6C) and MANF (Fig. 6D) in MMCs. Silencing CALR and MANF (Additional file 8: Fig. S5C and S5D) increased ER stress (Fig. 6E, F). Knockdown of CALR (Fig. 6G) or MANF (Fig. 6H) did not affect RCN3 expression in MMCs. Knockdown of CALR did not change the expression of MANF (Fig. 6G), and vice versa (Fig. 6H). RCN3 deficiency might suppress CALR and MANF expressions independently, suggesting CALR and MANF induced ER stress through an unconnected pathway.

### Inhibition of miR-92a-1-5p protected kidney function and ameliorated DN progression in db/db mice

**Fig. 7:**
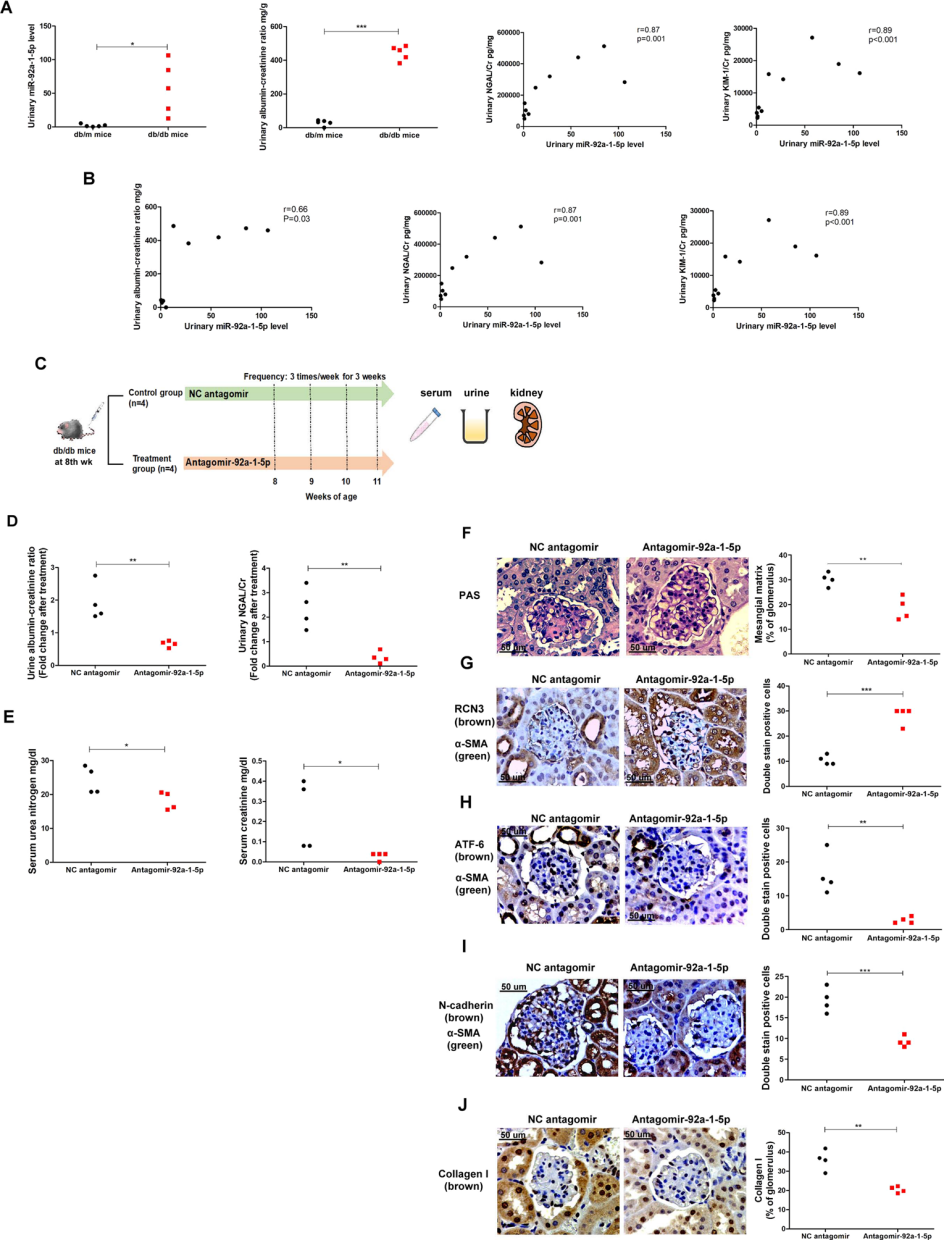
Inhibition of miR-92a-1-5p protected renal function in db/db diabetic mice and ameliorated diabetic nephropathy (DN) progression in vivo. **A** Urinary exosomal miR-92a-1-5p levels, albumin-creatinine ratio (ACR), neutrophil gelatinase-associated lipocalin/creatinine (NGAL/Cr) and kidney injury molecule 1/creatinine (KIM-1/Cr) expressions were measured in db/db mice (n = 5) and db/m mice (n = 5) at 12th week. **B** The correlation between urinary exosomal miR-92a-1-5p levels and urinary ACR, NGAL/Cr and KIM-1/Cr in mice at 12th week. **C** Flow chart of intervention study in vivo. **D** Urinary ACR and NGAL/Cr levels were measured in db/db mice treated with antagomir-92a-1-5p (n = 4) compared with those treated with negative control (NC) antagomir (n = 4). **E** Serum urea nitrogen (UN) and creatinine were examined in db/db mice treated with antagomir-92a-1-5p compared to those treated with NC antagomir. Exosomal miR-92a-1-5p in the urine of mice was isolated, and then assessed by qRT-PCR. Urine albumin was measured using immunoturbidimetric assay. Serum UN and urine creatinine levels were determined using the enzymatic method. Serum creatinine was measured using the compensated Jaffé (kinetic alkaline picrate) method. The concentration of NGAL and KIM-1 in urine was measured using enzyme-linked immunosorbent assay (ELISA). **F** Periodic acid-schiff stain of kidneys in mice. **G-I** The expressions of RCN3, ATF-6, and N-cadherin in MCs of kidneys in mice (NC antagomir (n = 4), antagomir-92a-1-5p (n = 4)). The kidney sections were co-stained with RCN3, ATF-6, and N-cadherin (brown) and α-SMA (green), as the marker of MCs. The images quantification was performed using the IHC Profiler Plugin of ImageJ Software. The bar graph represents the mean ± S.E.M. *p < 0.05, **p < 0.01, ***p < 0.001 by Student’s t-test. *p*-value of correlation was analyzed by Spearman analysis

Since exosomal miR-92a-1-5p secreted from PTECs contributed to MFT in MCs, we further examined the pathophysiologic role of miR-92a-1-5p in a mouse model. Urinary albumin-creatinine ratio (ACR), NGAL/Cr, KIM-1/Cr levels, and urinary miR-92a-1-5p, were elevated in db/db mice compared with db/m mice (Fig. 7A). Urinary exosomal miR-92a-1-5p level was positively correlated with urinary levels of ACR, Kim-1/Cr and NGAL/Cr (Fig. 7B), indicating that miR-92a-1-5p contributed to the changes in kidney function in DN.

Next, we hypothesized that miR-92a-1-5p inhibition may protect kidney function in db/db mice. A chemically modified antagomir-92a-1-5p was used to block endogenous miR-92a-1-5p function (Fig. 7C). Antagomir-92a-1-5p treatment significantly decreased urinary levels of ACR and NGAL/Cr in db/db mice compared to those treated with NC antagomir (Fig. 7D). Antagomir-92a-1-5p treatment not only improved serum UN and Cr levels (Fig. 7E), but also reduced mesangial matrix in glomerulus of db/db mice in PAS stain (Fig. 7F). Antagomir-92a-1-5p treatment increased the expression of RCN3 (Fig. 7G) and attenuated the levels of ATF-6 (Fig. 7H) and N-cadherin (Fig. 7I) in the glomeruli of db/db mice. These findings supported the inhibition of miR-92a-1-5p as a potential renoprotective strategy in diabetic mice.

### Urinary exosomal miR-92a-1-5p level as a biomarker of kidney injury in humans

**Fig. 8:**
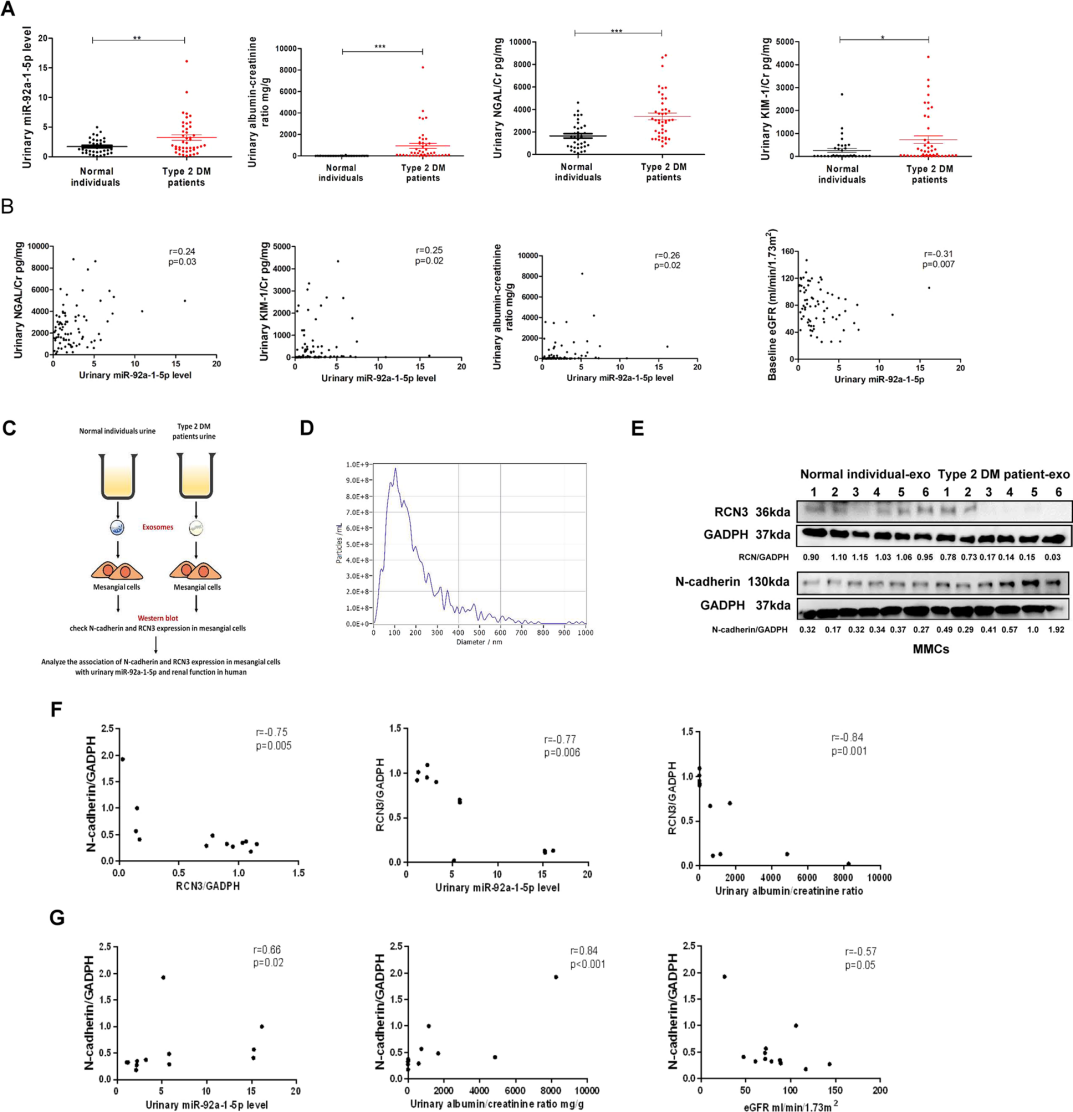
Urinary exosomal miR-92a-1-5p level as a biomarker of renal injury and a predictor of rapid decline in kidney function in humans. **A** Urinary exosomal miR-92a-1-5p, ACR, NGAL/Cr, and KIM-1/Cr levels were examined in type 2 diabetic (T2D) patients (n = 44) compared to healthy individuals (n = 36). **B** The association of urinary exosomal miR-92a-1-5p levels with urinary ACR, NGAL/Cr, and KIM-1/Cr, and estimated glomerular filtration rate (eGFR) in the participants. **C** The flow chart shows the experiment of investigating the effect of urinary exosomes on MMCs. MMCs were treated with urinary exosomes derived from normal individuals (n = 6) and type 2 diabetic patients (n = 6) for 48 h. **D** Size distribution plots of urinary exosomes in humans using nanoparticle tracking analysis. **E** N-cadherin and RCN3 levels were examined in MMCs treated with urinary exosomes derived from normal individuals and T2D patients at the same time. **F** The association of RCN3 expression with N-cadherin expression in MMCs, and urinary miR92-a-1-5p level and ACR in the participants. **G** The association of N-cadherin expression in MMCs with urinary miR92-a-1-5p level and urinary ACR, and baseline eGFR in the participants. Exosomal miR-92a-1-5p in the urine of mice was isolated, and then assessed by qRT-PCR. Urine albumin was measured using the immunoturbidimetric assay, and urine creatinine was determined using the enzymatic method. The levels of NGAL in urine were measured using Magnetic Luminex® Assay. The concentrations of KIM-1 in urine were measured using ELISA. Serum creatinine was measured using the compensated Jaffé (kinetic alkaline picrate) method. eGFR was calculated using the equation (eGFR = 186 × serum creatinine -1.154 × Age -0.203 × 0.742 (if female). The bar graph represents the mean ± S.E.M. *p < 0.05, **p < 0.01, ***p < 0.001 by Student’s t-test or ANOVA followed by the post hoc test with Tukey’s correction, and *p*-value of correlation was analyzed by Spearman analysis

miRNAs can be excreted into urine, and act as biomarkers for predicting DN progression [25]. We enrolled 36 normal individuals and 44 patients with T2D (Additional file 10: Table S7), and measured miR-92a-1-5p levels in urinary exosomes. T2D patients had higher urinary exosomal miR-92a-1-5p levels compared to the normal individuals (Fig. 8A). Urinary ACR, NGAL/Cr and KIM-1/Cr levels were also elevated in the T2D patients (Fig. 8A), and were positively correlated with urinary miR-92a-1-5p levels (Fig. 8B). More importantly, high urinary exosomal miR-92a-1-5p levels were significantly associated with low eGFR (r = − 0.31, *p* = 0.007, Fig. 8B).

Furthermore, we assessed the effect of exosomal miR-92a-1-5p isolated from the urine of healthy individuals and T2D patients in the induction MFT of MCs (Fig. 8C). The average diameter of urinary exosomes in human was 189.3 ± 140.9 nm (Fig. 8D). The results showed decreased RCN3 and elevated N-cadherin expressions in MMCs treated with urinary exosomes isolated from the T2D patients (Fig. 8E). RCN3 expression was negatively correlated with the N-cadherin expression in MMCs after urinary exosome treatment (Fig. 8F). The effects of urinary exosomes on RCN3 down-regulation (Fig. 8F) and N-cadherin up-regulation (Fig. 8G) were positively correlated with exosomal miR-92a-1-5p level and urinary ACR. Moreover, N-cadherin expression in MMCs was negatively associated with eGFR (Fig. 8G). Urinary exosomal miR-92a-1-5p could be a biomarker of kidney injury in clinical T2D patients.

## Discussion

In this cross-disciplinary study, we found that exosomal miR-92a-1-5p derived from HG-treated HK-2 cells induced pathophysiologic injury in the glomerulus microenvironment, resulting in the DN development. Exosomes are important signals mediators and responsible for the communication among various kidney cells [25–28]. Exosomal biomaterials released from different kidney cells to urine may carry valuable information for stage-specific prognosis [12, 29, 30]. The present study demonstrated that communication between proximal tubules and glomeruli modulated the kidney microenvironment via exosomal miR-92a-1-5p derived from PTECs in vivo and in vitro models, which altered the phenotype in MCs resulting in DN progression.

**Fig. 9:**
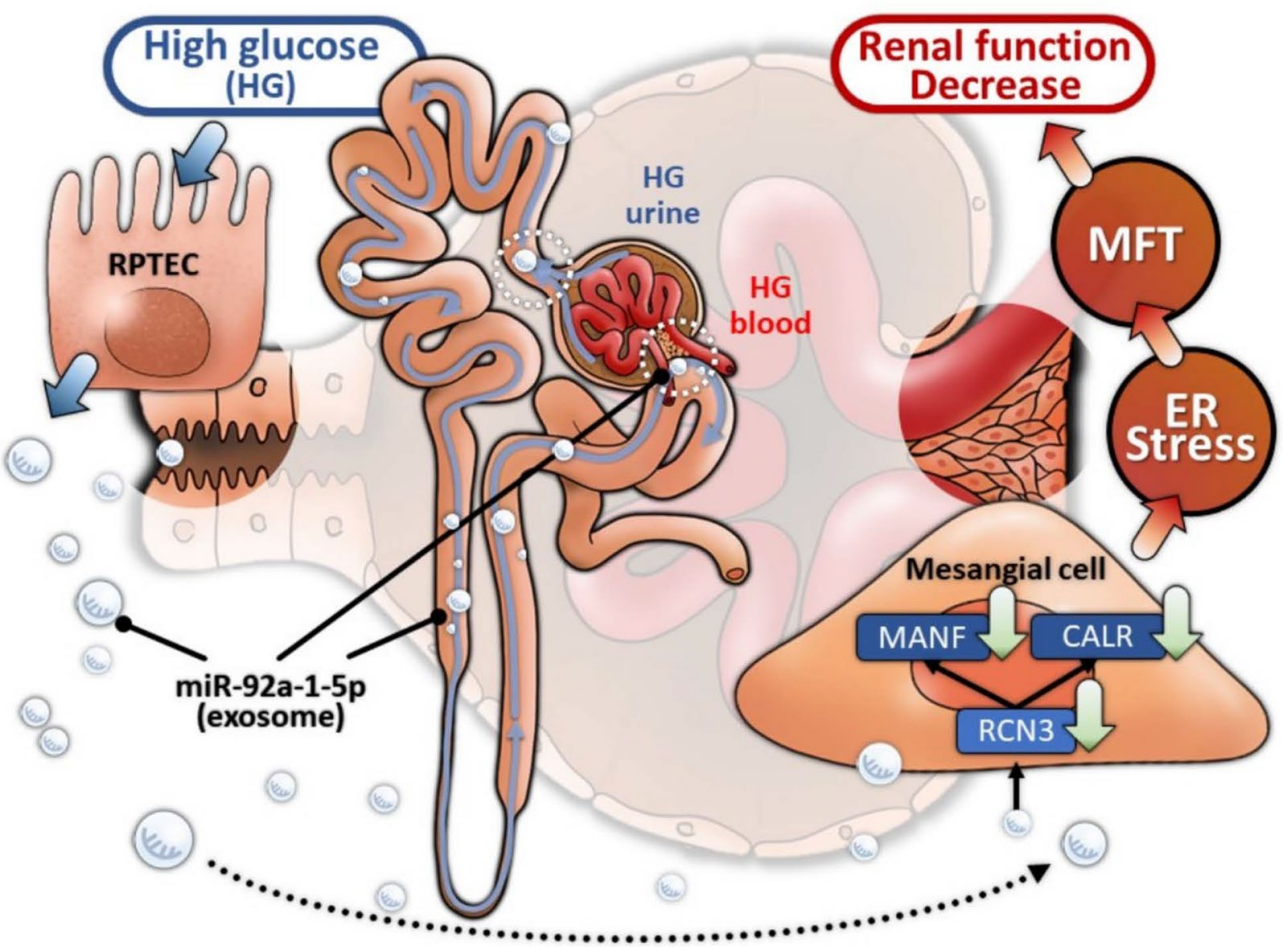
Illustration of the mechanism of PTEC-derived exosomal miR-92a-1-5p in DN. HG increased miR-92a-1-5p expression in PTECs, which in turn induced ER stress and MFT in MCs through exosome delivery targeting RCN3 in DN

miR-92a has been known as an oncomir by increasing cancer growth and epithelial-mesenchymal transition (EMT) in several cancers [31–33]. In addition, miR-92a was correlated with CKD-induced uremia, oxidative stress, and endothelial dysfunction [34], and miR-92a upregulation impaired endothelial function in db/db mice [35]. We firstly identified the unique role of miR-92a-1-5p in the cross-talk between PTECs and MCs in DN development. miR-92a-1-5p carried by PTECs-derived exosomes induced ER stress, glomerular matrix expansion and MFT in MCs, and urinary exosomal miR-92a-1-5p could be a clinical marker of kidney injury. More importantly, miR-92a-1-5p antagomir ameliorated renal injuries from structure to bio-function in DN mouse model. This proves the impact of exosomal miR-92a-1-5p on cross-talk with kidney cells in pathophysiologic mechanisms and suggests its potential use in clinical care of DN (Fig. 9).

The excessive deposition of extracellular matrix proteins and MFT in the MCs and EMT in tubulointerstitium are the landmarks of DN [20]. ER stress could promote MFT and be involved in the development and progression of DN [21, 36, 37]. Several studies have reported that ER stress promoted EMT in HK-2 cells [38, 39] and inhibiting ER stress in HK-2 cells could attenuate MC-derived MFT [40]. One of our unique findings showed that PTEC-derived exosomal miR-92a-1-5p was transmitted to MCs and caused ER stress resulting in MFT in MCs through modulating RCN3 expression. RCN3 is an ER lumen protein and its deficiency has been reported to exacerbate pulmonary fibrosis [41]. Current study demonstrated that RCN3 knockdown promoted ER stress and MFT, and conversely, RCN3 overexpression ameliorated ER stress and MFT induced by HG-derived exosomes and miR-92a-1-5p in MCs. We further found that CALR and MANF, both of which are ER stress inducible proteins, were involved in RCN3 deficiency-induced ER stress process, and either CALR or MANF deficiency resulted in ER stress in MCs of DN. Moreover, RCN3 modulated CALR and MANF expressions individually. The independent signal transmissions of RCN3-CALR and RCN3-MANF may be related to different bio-functions between CALR and MANF in ER. CALR as a Ca^2+^-binding chaperone located in ER lumen is responsible for modulating proper folding of neo-synthesized glycoproteins and for calcium retention [42, 43]; however, MANF as a negative regulator of unfolded protein response could alleviate hepatocellular carcinoma by suppressing EMT [44, 45]. MANF not only participated in ER stress-related kidney disease [46, 47], but also rescued acute kidney injury [48]. Our findings validated the pathophysiologic role of CALR and MANF in DN, and provided a novel signal cascade “miR-92a-1-5p-RCN3-CALR/MANF” for the progression of DN.

Glomerulopathy in DN occurs as a consequence of the interactions within kidney, including glomerular podocytes, mesangial cells and endothelial cells. Accumulating evidences indicate proximal tubule might promote glomerulopathy in kidney diseases through retrograde trafficking [7, 8]. Hasegawa et al. demonstrated that PTECs-derived nicotinamide mononucleotide diffused back to the glomerulus, and triggered podocyte foot process effacement, further leading to proteinuria and glomerulosclerosis [7]. Other studies also revealed that proximal tubular injury induces not only podocytopathy but also more extensive glomerular injury [9]. Exosomes, an important mediator of cell–cell interaction within organs, might be released into the extracellular space, and travel in the interstitial fluid to neighbouring cells, or in the circulation to distant targets [49]. Therefore, further study is required to explore the transmission tract from PTECs-derived exosomal miR-92a-1-5p to glomerulus.

In conclusion, exosomal miR-92a-1-5p derived from PTECs under HG induced ER stress and MFT in MCs through targeting RCN3. The cross-talk between PTECs and MCs by exosomes contributed to DN progression. Blocking miR-92a-1-5p epigenetic regulatory network may be a potential therapeutic strategy for DN.

## Supplementary Information

**Additional file 1: Fig. S1.** **A** Identify primary proximal tubular epithelial cell (PTECs) and mesangial cell (MCs), human kidney-2 (HK-2) cells, and mouse MCs, by aquaporin 1 (AQP1) and α-smooth muscle actin (α-SMA) using western blotting. **B** The efficiency of injection via tail vein. **C** Liver function after mir-92a-1-5p treatment in vivo model (n=4).

**Additional file 2: Table S1.** Target sequence of materials utilized in the study.

**Additional file 3: Table S2.** The list of reagents in the study.

**Additional file 4: Fig. S2.** The detection of exosomes and the effects of different doses of proximal tubular epithelial cells (PTECs)-derived exosomes on mesangial cells (MCs). **A** Size distribution plots of HK-2 cells-derived exosomes under NG or HG using nanoparticle tracking analysis. **B** The expression of CD63, as a marker of exosomes, in kidneys of mice and humans using immunohistochemistry stain. **C** Surface markers of exosomes were examined using western blot. **D** The effect of different doses of exosomes derived from HK-2 cells on E-cadherin, N-cadherin and vimentin expression in mouse MCs at 48 h using western blotting. *p<0.05, **p<0.01, ***p<0.001 by ANOVA followed by the post hoc test adjusted with Tukey’s correction.

**Additional file 5: Fig. S3.** Bioinformatics analysis of miRNAs from renal proximal tubular epithelial cells (RPTECs). **A** Flowchart of identification of potential miRNAs from RPTECs obtained from a normal individual and a type 2 diabetic patient by next generation sequencing (NGS) and following bioinformatics analysis. **B** The heat map revealed differentially expressed miRNAs from normal and diabetic PTECs with log2(seq) values. **C** Tox analysis of miR-92a-1-5p regulatory targets according to IPA core analysis.

**Additional file 6: Table S3.** Cellular component analysis of predicted targets of miR-92a-1-5p according to DAVID database. **Table S4.** Cellular component analysis of predicted targets of miR-92a-1-5p according to STRING database. **Table S5.** miR-92a-1-5p targeted RCN3 according to miRNA target filter analysis of IPA database.

**Additional file 7: Fig. S4.** Bioinformatics analysis of RCN3. **A** The predictive binding score of miR-92a-1-5p on 3’UTR of RCN3 mRNA according to miRmap database. **B** A schematic representation of sequence alignment of RCN3 mRNA 3’UTR based on TargetScan version 7.1. **C**–**E** Ontology analysis of cellular component, KEGG pathway, and biologic process of these dysregulated genes in MMCs transfected with RCN3 siRNA are displayed in the pie chart according to DAVID database. The numbers that are shown outside each pie segment indicates the number of genes involved in each term.

**Additional file 8: Fig. S5.** **A** The efficiency of RCN3 suppression. **B** The efficiency of RCN3 overexpression. **C, D** The efficiency of CALR and MANF suppression in mouse mesangial cells after transfection of RCN3 siRNA, RCN3 cDNA, CALR siRNA and MANF siRNA respectively

**Additional file 9: Table S6.** KEGG or Cellular component analysis of silencing RCN3 according to DAVID database.

**Additional file 10: Table S7.** The clinical characteristics of human participates.

## Data Availability

The datasets generated and/or analyzed during the current study are available from the corresponding author on reasonable request.

## Abbreviations

ACR: Albumin-creatinine ratio
ATF6: Activating transcription factor 6
CALR: Calreticulin
CHOP: C/EBP homologous protein
CKD: Chronic kidney disease
CM: Conditional medium
DAVID: Database for annotation, visualization and integrated discovery
DM: Diabetes mellitus
DN: Diabetic nephropathy
eGFR: Estimated glomerular filtration rate
ER: Endoplasmic reticulum
ESRD: End stage renal disease
EV: Extracellular vesicular
EVs: Extracellular vesicles
HEK: Human embryonic kidney
HG: High glucose
HK-2: Human kidney-2
HSP70: Heat shock proteins 70
IHC: Immunohistochemistry stain
IPA: Ingenuity pathway analysis
IRE1α: Inositol-requiring transmembrane kinase/endoribonuclease 1α
KIM-1: Kidney injury molecule 1
MANF: Mesencephalic astrocyte derived neurotrophic factor
MFT: Myofibroblast transdifferentiation
MC: Mesangial cell
miRNA: MicroRNA
NG: Normal glucose
NGAL: Neutrophil gelatinase-associated lipocalin
NGS: Next generation sequencing
NTA: Nanoparticle tracking analysis
PBS: Phosphate-buffered saline
PERK: Protein kinase r-like endoplasmic reticulum kinase
PTEC: Proximal tubular epithelial cell
RCN3: Reticulocalbin-3
RIPA: Radio-immunoprecipitation assay
RT-Q-PCR: Reverse transcription and quantitative real time polymerase chain reaction TBS: Tris-Buffered Saline
TEM: Transmission electron microscope
TSG101: Tumor susceptibility gene 101
3′UTR: 3′-Untranslated region
UTUC: Upper tract urothelial carcinoma
