# Supplementary figures and images for: Proximal tubule-derived exosomes contribute to mesangial cell injury in diabetic nephropathy via miR-92a-1-5p transfer
Source: Cell Commun Signal. 2023 Jan 13;21:10. doi: 10.1186/s12964-022-00997-y (PMC9838003; doi:10.1186/s12964-022-00997-y)

**A**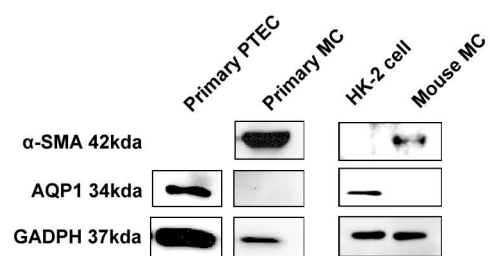**B**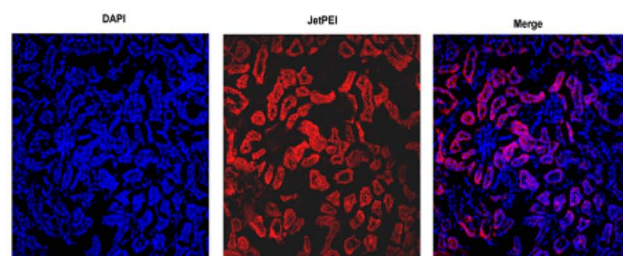**C**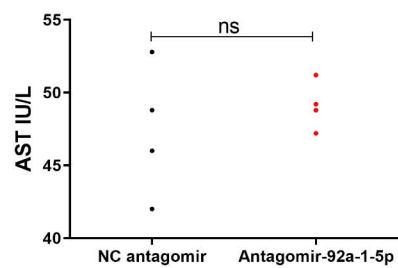

Supplement: Supplementary file 2 — Additional file 1: Fig. S1. A Identify primary proximal tubular epithelial cell (PTECs) and mesangial cell (MCs), human kidney-2 (HK-2) cells, and mouse MCs, by aquaporin 1 (AQP1) and α-smooth muscle actin (α-SMA) using western blotting. B The efficiency of injection via tail vein. C Liver function after mir-92a-1-5p treatment in vivo model (n=4). [file 12964_2022_997_MOESM2_ESM.pdf]

**A**

HK-2 cells treated with NG

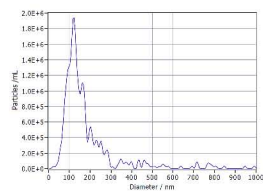

HK-2 cells treated with HG

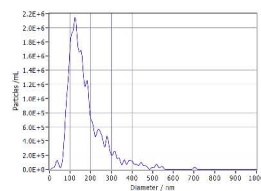**B**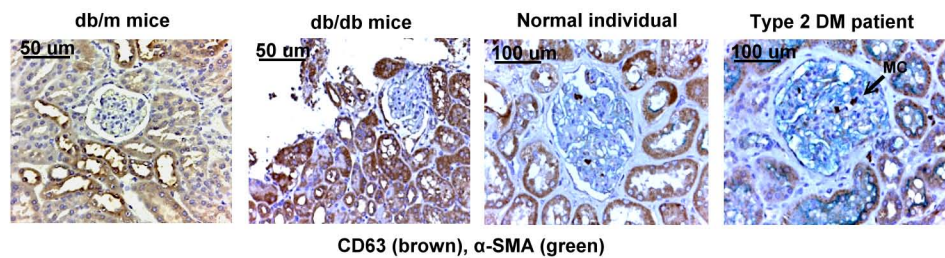**C**exosomes  
in the urine of mice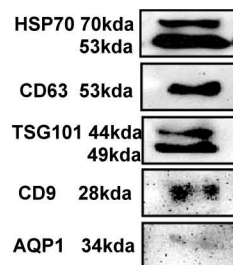**D**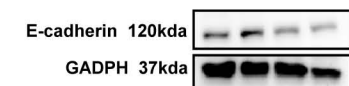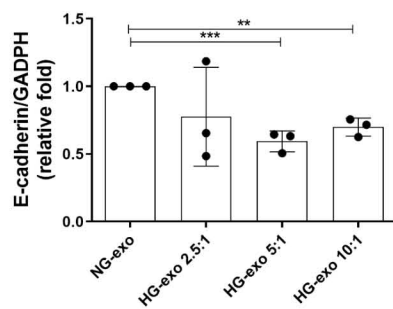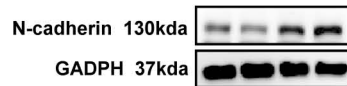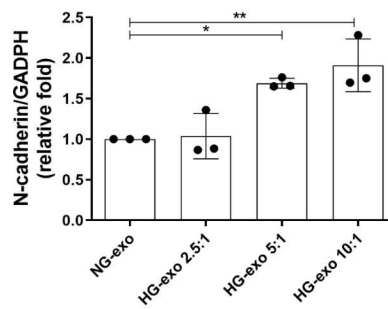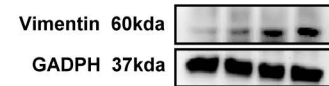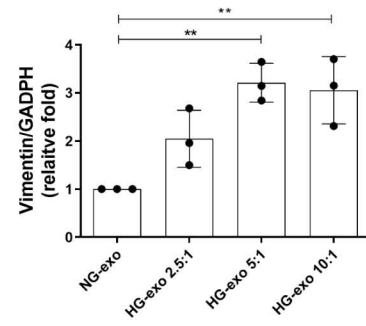

Supplement: Supplementary file 5 — Additional file 4: Fig. S2. The detection of exosomes and the effects of different doses of proximal tubular epithelial cells (PTECs)-derived exosomes on mesangial cells (MCs). A Size distribution plots of HK-2 cells-derived exosomes under NG or HG using nanoparticle tracking analysis. B The expression of CD63, as a marker of exosomes, in kidneys of mice and humans using immunohistochemistry stain. C Surface markers of exosomes were examined using western blot. D The effect of different doses of exosomes derived from HK-2 cells on E-cadherin, N-cadherin and vimentin expression in mouse MCs at 48 h using western blotting. *p<0.05, **p<0.01, ***p<0.001 by ANOVA followed by the post hoc test adjusted with Tukey’s correction. [file 12964_2022_997_MOESM5_ESM.pdf]

**A**

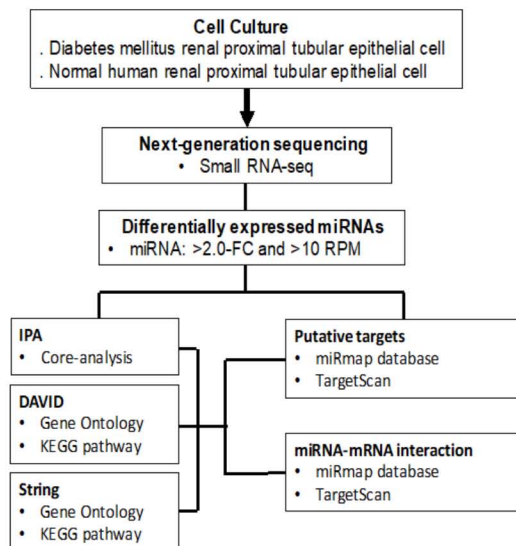

**B**

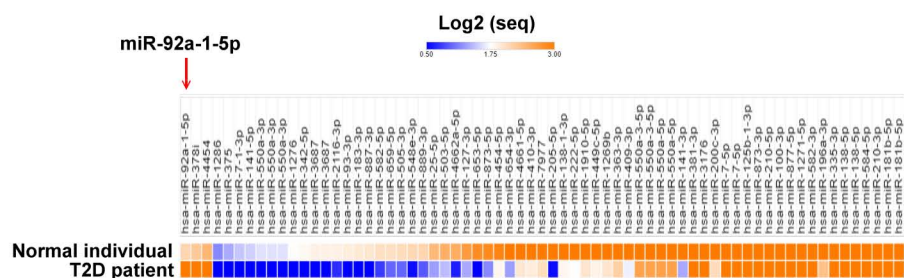

**C**

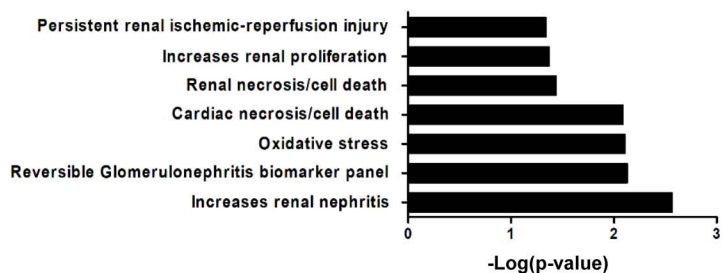

**Fig S3**

Supplement: Supplementary file 6 — Additional file 5: Fig. S3. Bioinformatics analysis of miRNAs from renal proximal tubular epithelial cells (RPTECs). A Flowchart of identification of potential miRNAs from RPTECs obtained from a normal individual and a type 2 diabetic patient by next generation sequencing (NGS) and following bioinformatics analysis. B The heat map revealed differentially expressed miRNAs from normal and diabetic PTECs with log2(seq) values. C Tox analysis of miR-92a-1-5p regulatory targets according to IPA core analysis. [file 12964_2022_997_MOESM6_ESM.pdf]

**A**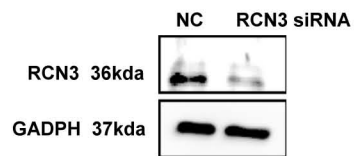**B**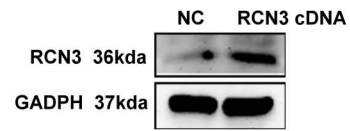**C**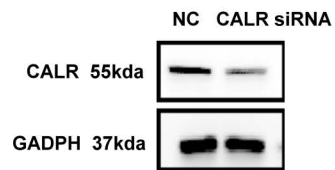**D**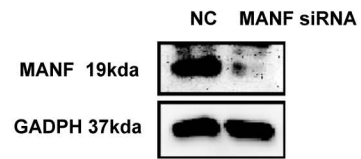

Supplement: Supplementary file 9 — Additional file 8: Fig. S5. A The efficiency of RCN3 suppression. B The efficiency of RCN3 overexpression. C, D The efficiency of CALR and MANF suppression in mouse mesangial cells after transfection of RCN3 siRNA, RCN3 cDNA, CALR siRNA and MANF siRNA respectively [file 12964_2022_997_MOESM9_ESM.pdf]
