# Supplementary material for: Proximal tubule-derived exosomes contribute to mesangial cell injury in diabetic nephropathy via miR-92a-1-5p transfer
Source: Cell Commun Signal. 2023 Jan 13;21:10. doi: 10.1186/s12964-022-00997-y (PMC9838003; doi:10.1186/s12964-022-00997-y)
Supplement: Supplementary file 3 — Additional file 2: Table S1. Target sequence of materials utilized in the study. [file 12964_2022_997_MOESM3_ESM.pdf]

Table S1. Target sequence of materials utilized in the study

| description | Mature microRNA sequence |
|---|---|
| ON-TARGETplus Mouse RCN3(52377) siRNA | Target Sequence:<br>ACGAUGACGCCCACGGGAA<br>GUUGGGAGGAGUUGCGCAA<br>CAUCCUGACCUAACCGCAA<br>GGGAAGAGCUGACGGCCUU |
| ON-TARGETplus CALR siRNA | Target Sequence:<br>CCUAUGAGGUGAAGAUUGA<br>GCACGGAGACUCAGAAUAC<br>GAAGCUGUUUCCUAAUAGU<br>GCAAGGAUGAUGAGUUUAC |
| ON-TARGETplus MANF siRNA | Target Sequence:<br>UGAAGAAGCUCCGAGUUAA<br>GAGAAUCGGUUGUGCUACU<br>GACUACAUCCGGAAGAUAA<br>CGACUGCGAAGUUUGUAUU |
| ON-TARGETplus Non-targeting | Target Sequence:<br>UGGUUUACAUGUCGACUAA<br>UGGUUUACAUGUUGUGUGA<br>UGGUUUACAUGUUUUCUGA<br>UGGUUUACAUGUUUUCCUA |

| | |
|---|---|
| miRIDIAN microRNA Human has-miR-92a-1-5p-Mimic | Mature microRNA sequence<br>AGGUUGGGAUCGGUUGCAAUGCU |
| miRIDIAN microRNA Human has-miR-92a-1-5p-Hairpin Inhibitor | Mature microRNA sequence<br>AGGUUGGGAUCGGUUGCAAUGCU |
| miRIDIAN microRNA mmu-miR-92a-1-5p-Hairpin Inhibitor | Mature microRNA sequence<br>AGGUUGGGAUUUGUCGCAAUGCU |
