# Supplementary material for: Proximal tubule-derived exosomes contribute to mesangial cell injury in diabetic nephropathy via miR-92a-1-5p transfer
Source: Cell Commun Signal. 2023 Jan 13;21:10. doi: 10.1186/s12964-022-00997-y (PMC9838003; doi:10.1186/s12964-022-00997-y)
Supplement: Supplementary file 4 — Additional file 3: Table S2. The list of reagents in the study. [file 12964_2022_997_MOESM4_ESM.pdf]

Table S2. The list of reagents in the study

Protein	Catalog number
**BD Biosciences**	
N-Cadherin	Cat.610921
E-Cadherin	Cat.610182
Vimentin	Cat.550513
**Cell Signaling Technology**	
ATF-6	Cat.65880
PERK	Cat.3192
IRE1α	Cat.3294
CHOP	Cat.2895
Calreticulin	Cat.12238
**ThermoFisher scientific**	
Phospho-PERK	Cat.PA540294
Phospho-IRE1 alpha	Cat. PA1-16927
RCN3	Cat. PA5-98793
**Bethyl Laboratories**	
MANF	Cat. A305-572A
**EMD Millipore**	
GAPDH	Cat. mab374
**System Biosciences**	
Exosome Antibodies : CD9, CD63, CD81, Hsp70	Cat. EXOAB-KIT-1-SBI

**Abcam**	
TSG101	Cat.30871
**IHC antibody**	**Catalog number**
**Abcam**	
N-Cadherin	Cat.ab76057
Alpha-smooth muscle actin(α-sma)	Cat.ab5694
**ThermoFisher scientific**	
CD63	Cat.PA5-92370
RCN3	Cat.PA5-98793
**Sigma-Aldrich**	
ATF6	**Cat.PRS3681**
**Other agents**	
Keratinocyte serum free medium	Cat. 17005-042
ExoQuick-TC	Cat. EXOTC50A-1
Urine Exosome RNA Isolation Kit	Cat. 47200
Lipofectamine^{TM} RNAiMAX transfection reagent	Cat. 13778075
DharmaFECT Duo Transfection Reagent	Cat. T-2010-03
KIM-1 ELISA kit	Cat: MKM100
NGAL Magnetic Luminex® Assay	Cat: LXSAHM
