# Supplementary material for: Proximal tubule-derived exosomes contribute to mesangial cell injury in diabetic nephropathy via miR-92a-1-5p transfer
Source: Cell Commun Signal. 2023 Jan 13;21:10. doi: 10.1186/s12964-022-00997-y (PMC9838003; doi:10.1186/s12964-022-00997-y)
Supplement: Supplementary file 7 — Additional file 6: Table S3. Cellular component analysis of predicted targets of miR-92a-1-5p according to DAVID database. Table S4. Cellular component analysis of predicted targets of miR-92a-1-5p according to STRING database. Table S5. miR-92a-1-5p targeted RCN3 according to miRNA target filter analysis of IPA database. [file 12964_2022_997_MOESM7_ESM.pdf]

Table S3. Cellular component analysis of predicted targets of miR-92a-1-5p according to DAVID database

**Term**	**Count**	**p-value**	**Genes**
Plasma membrane	91	0.06	TLN1, GLDN, IL6ST, RAB5C, TLN2, WNT3A, SYT2, GLRA3, CD151, TNFSF18, IQGAP1, AQP2, FCRL3, TRIM4, ATP2B2, ATP2B4, WNT3, ANK1, UNC5B, SV2B, FCGR3A, RHOF, FCGR3B, USP14, CEACAM1, ISLR2, CLNS1A, EGFR, RAP2B, PRKCA, STX1A, MDGA1, VIL1, RIMBP2, SPINT1, PTPRT, NFAM1, PALLD, THY1, PJA2, CD38, JOSD1, KLRG1, VSIG1, ANXA8L1, CHRM2, CLDN2, CNTN4, WASL, CLIP3, CHL1, SLC38A5, RALGPS2, NKD1, ALPI, GSDMA, CATSPERD, NPY2R, PPP1R12B, BDKRB2, OCRL, IL7R, GPRC5A, TJAP1, CDH5, ANXA8, TMEM127, GPM6A, SERINC1, PPL, CNR1, SRR, VMP1, MR1, EHD1, SCN5A, ENTPD2, GNAT1, SLCO4C1, NF2, EPB42, IL1RN, SIRT2, GCG, NMT1, CSNK1D, PRLR, RAB22A, CHRNB2, CIT, HTR2C
Extracellular exosome	70	0.01	EIF6, ARSB, SRP14, TLN1, COPS6, IL6ST, RAB5C, WNT3A, FSTL1, LUZP1, IQGAP1, AQP2, ST3GAL1, ATP2B2, WNT3, TPP1, FCGR3A, CTBS, RHOF, FCGR3B, USP14, CEACAM1, KLK13, MB, PRKCA, RAP2B, CHTF8, VIL1, SPINT1, GLTP, EML5, THY1, CD38, PA2G4, ANXA8L1, TXNDC5, CLDN2, KPNA4, WASL, UGGT1, WFDC2, CHL1, YWHAZ, LMAN2, OCRL, TIMP3, GPRC5A, GALM, PFN2, HNRNPK, GPM6A, SERINC1, DDX19B, PPL, SYAP1, NAGA, KRT2, ANGPTL1, EHD1, IVL, ENTPD2, DPT, NAT8, SLCO4C1, IL1RN, MYO1D, CREB5, SH3BP4, SRSF7, RAB22A
Cell surface	18	0.02	ARSB, EGFR, TLN1, PPFIA4, WNT3A, PTPRT, NFAM1, LMAN2, TNFSF18, CDH5, CD38, PRLR, VAMP4, SCN5A, CEACAM1, USP14, ISLR2, SPTB
Cell junction	16	0.02	DLGAP1, STX1A, GLRA3, SYT2, RIMBP2, FARP1, CDH5, PJA2, ATP2B2, NMT1, CBLN1, CHRM2, C4ORF19, CHRNB2, SV2B, CEACAM1
Focal adhesion	13	0.06	EGFR, YWHAZ, TLN1, TLN2, CD151, PALLD, ARHGAP26, IQGAP1, THY1, ATAT1, HNRNPK, STARD8, LMLN

Actin cytoskeleton	11	0.008	NMT1, CORO2A, TLN2, PPP1R12B, RAB22A, CORO6, WIPF1, WASL, PALLD, IQGAP1, SPTB
Neuronal cell body	11	0.07	GNAT1, ATP2B2, GPM6A, CHRM2, RBM8A, IL6ST, MYO1D, SRR, SLC25A27, CIT, PPARGC1A
Cell-cell adherens junction	11	0.08	EGFR, TLN1, YWHAZ, HNRNPK, PPME1, CSNK1D, PPL, ASAP1, EHD1, GPRC5A, IQGAP1
Ruffle	9	3.06E-04	TLN1, NF2, TLN2, VIL1, RAB22A, WIPF1, PALLD, FGD6, IQGAP1
Growth cone	9	0.001	CCDC120, CNR1, TMOD2, IGF2BP1, DPYSL3, PALLD, IQGAP1, SIRT2, THY1
synapse	9	0.02	SHISA6, EGFR, ATP2B2, DLGAP1, PPFIA4, TLN2, CHRM2, RIMBP2, USP14
Lamellipodium	8	0.03	NF2, ARHGEF6, VIL1, IGF2BP1, DPYSL3, WASL, PALLD, FGD6
Endoplasmic reticulum lumen	8	0.07	ARSB, GCG, WNT3, TXNDC5, WNT3A, COL1A1, **RCN3**, UGGT1
Membrane raft	8	0.09	RAP2B, EGFR, UNC5B, CNR1, CLIP3, NFAM1, IQGAP1, THY1

Table S4. Cellular component analysis of predicted targets of miR-92a-1-5p according to STRING database

**Pathway description**	**Observed gene count**	**False discovery rate**	**Matching proteins in the network**
Endoplasmic reticulum lumen	8	7.48E-14	ARSB,COL1A1,GCG,**RCN3**,TXNDC5,UGGT1,WNT3,WNT3A
Endoplasmic reticulum	7	3.92E-05	COL1A1,GCG,**RCN3**,TXNDC5,UGGT1,WNT3,WNT3A
Extracellular region part	7	0.006	ARSB,COL1A1,GCG,TXNDC5,UGGT1,WNT3,WNT3A
Extracellular region	7	0.01	ARSB,COL1A1,GCG,TXNDC5,UGGT1,WNT3,WNT3A
Endomembrane system	6	0.04	GCG,**RCN3**,TXNDC5,UGGT1,WNT3,WNT3A
Proteinaceous extracellular matrix	3	0.03	COL1A1,WNT3,WNT3A
Lysosomal lumen	2	0.04	ARSB,TXNDC5

Table S5. miR-92a-1-5p targeted RCN3 according to miRNA target filter analysis of IPA database

miRNA	Precursor	Log2 Ratio	Target Gene	Log2 Ratio
hsa-miR-92a-1-5p	hsa-mir-92a-1	1.05	RCN3	-2.332
