# Supplementary material for: Proximal tubule-derived exosomes contribute to mesangial cell injury in diabetic nephropathy via miR-92a-1-5p transfer
Source: Cell Commun Signal. 2023 Jan 13;21:10. doi: 10.1186/s12964-022-00997-y (PMC9838003; doi:10.1186/s12964-022-00997-y)
Supplement: Supplementary file 8 — Additional file 7: Fig. S4. Bioinformatics analysis of RCN3. A The predictive binding score of miR-92a-1-5p on 3’UTR of RCN3 mRNA according to miRmap database. B A schematic representation of sequence alignment of RCN3 mRNA 3’UTR based on TargetScan version 7.1. C–E Ontology analysis of cellular component, KEGG pathway, and biologic process of these dysregulated genes in MMCs transfected with RCN3 siRNA are displayed in the pie chart according to DAVID database. The numbers that are shown outside each pie segment indicates the number of genes involved in each term. [file 12964_2022_997_MOESM8_ESM.pdf]

A

mRNA	Gene	Probability exact	Consevation phylop	miRmap score
hsa-miR-92a-1-5p	RCN3	93.46	87.67	90.31

B

hsa-miR-92a-1-5p 3'............GUUGGCUAGGGUUGGA...5'

nt(289-295)

WT RCN3 3'UTR human 5'............GAACCGC - CCCAACCC....3'
mouse 5'..................................CCAACCCU....3'
rabbit 5'..................................CCUCCCAU....3'

C

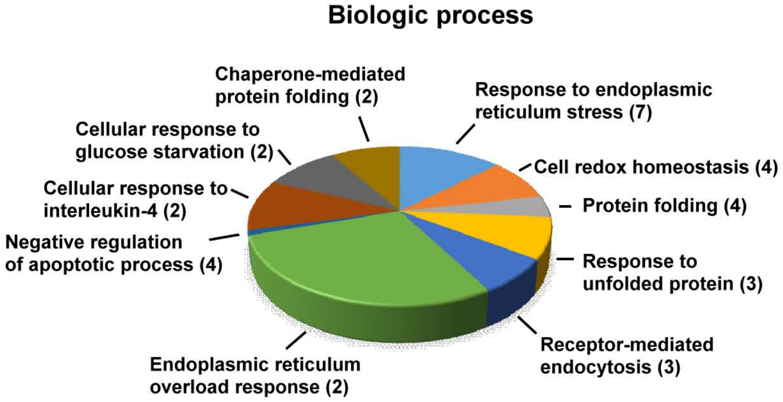

D

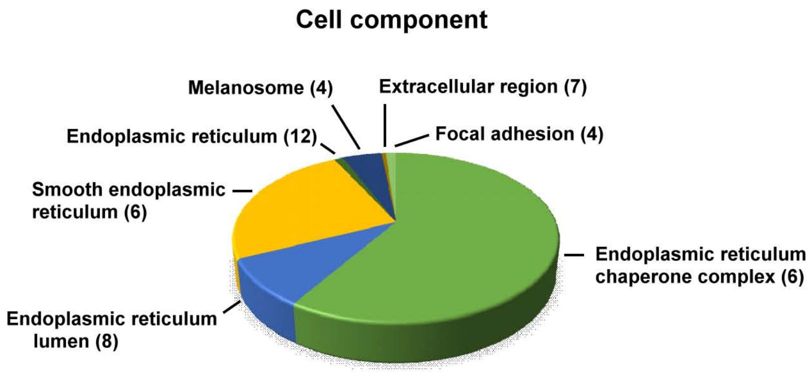

E

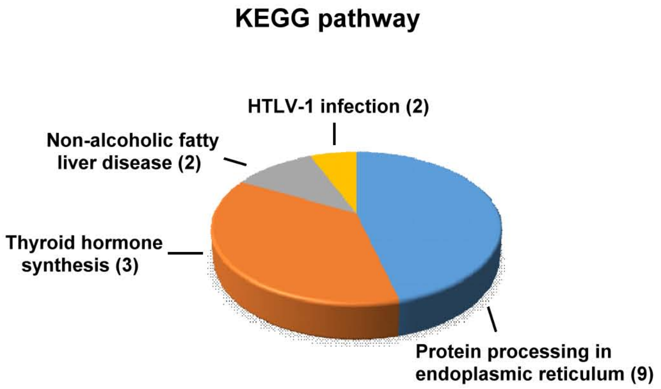

Fig S4
