# Supplementary material for: Proximal tubule-derived exosomes contribute to mesangial cell injury in diabetic nephropathy via miR-92a-1-5p transfer
Source: Cell Commun Signal. 2023 Jan 13;21:10. doi: 10.1186/s12964-022-00997-y (PMC9838003; doi:10.1186/s12964-022-00997-y)
Supplement: Supplementary file 10 — Additional file 9: Table S6. KEGG or Cellular component analysis of silencing RCN3 according to DAVID database. [file 12964_2022_997_MOESM10_ESM.pdf]

Table S6. KEGG or Cellular component analysis of silencing RCN3 according to DAVID database

**KEGG pathway**			
Term	Count	p-value	Genes
Protein processing in endoplasmic reticulum	9	1.90E-12	HYOU1, ERO1LB, HSP90B1, XBP1, PDIA6, PDIA4, HSPA5, **CALR**, DDIT3
Thyroid hormone synthesis	3	0.003	HSP90B1, PDIA4, HSPA5
Non-alcoholic fatty liver disease (NAFLD)	2	0.18	XBP1, DDIT3
HTLV-I infection	2	0.30	XBP1, CALR

**Cellular component**			
Term	Count	p-value	Genes
Endoplasmic reticulum chaperone complex	6	3.75E-13	HYOU1, HSP90B1, SDF2L1, PDIA6, PDIA4, HSPA5
Endoplasmic reticulum lumen	8	4.51E-12	HYOU1, HSP90B1, SDF2L1, PDIA6, PDIA4, HSPA5, **CALR, RCN3**
Smooth endoplasmic reticulum	6	5.56E-11	HYOU1, HSP90B1, PDIA6, PDIA4, HSPA5, **CALR**
Endoplasmic reticulum	12	5.62E-09	HYOU1, ERO1LB, HSP90B1, CRELD2, XBP1, SDF2L1, PDIA6, PDIA4, HSPA5, **CALR, RCN3, MANF**
Melanosome	4	1.20E-04	HSP90B1, PDIA6, PDIA4, HSPA5
Extracellular region	7	0.004	HYOU1, HSP90B1, CRELD2, HSPA5, **CALR**, CSN3, **MANF**
Focal adhesion	4	0.006	HYOU1, HSP90B1, HSPA5, **CALR**
Cell surface	4	0.02	CDH17, PDIA4, HSPA5, **CALR**
Endoplasmic reticulum membrane	4	0.03	ERO1LB, HSP90B1, SDF2L1, HSPA5
Extracellular matrix	3	0.03	HSP90B1, HSPA5, **CALR**
