# Supplementary material for: Proximal tubule-derived exosomes contribute to mesangial cell injury in diabetic nephropathy via miR-92a-1-5p transfer
Source: Cell Commun Signal. 2023 Jan 13;21:10. doi: 10.1186/s12964-022-00997-y (PMC9838003; doi:10.1186/s12964-022-00997-y)
Supplement: Supplementary file 11 — Additional file 10: Table S7. The clinical characteristics of human participates. [file 12964_2022_997_MOESM11_ESM.pdf]

Table S7. The clinical characteristics of human participates

	Normal Individuals N = 36	Type 2 Diabetes N = 44	p-value
Age, years	62.1 ± 6.2	65.2 ± 11.0	0.11
Sex (male), %	38.9	59.1	0.07
Fasting blood glucose, mg/dL	94.1 ± 10.0	144.3 ± 49.5	<0.001
Blood urea nitrogen, mg/dL	14.8 ± 2.7	23.7 ± 10.5	<0.001
Estimated glomerular filtration rate, mL/min/1.73m^2	96.3 ± 21.8	61.6 ± 31.0	<0.001
Urine albumin/creatinine ratio, mg/g	4.1 (3.0,6.4)	269.5 (59.3,1116.7)	<0.001

Data are expressed as number (percentage) for categorical variables and median (25th, 75th percentile) for continuous variables, as appropriate
